# Proteomic signatures improve risk prediction for common and rare diseases

**DOI:** 10.1038/s41591-024-03142-z

**Published:** 2024-07-22

**Authors:** Julia Carrasco-Zanini, Maik Pietzner, Jonathan Davitte, Praveen Surendran, Damien C. Croteau-Chonka, Chloe Robins, Ana Torralbo, Christopher Tomlinson, Florian Grünschläger, Natalie Fitzpatrick, Cai Ytsma, Tokuwa Kanno, Stephan Gade, Daniel Freitag, Frederik Ziebell, Simon Haas, Spiros Denaxas, Joanna C. Betts, Nicholas J. Wareham, Harry Hemingway, Robert A. Scott, Claudia Langenberg

**Affiliations:** 1 Human Genetics and Genomics, GSK Research and Development, Stevenage, UK; 2 MRC Epidemiology Unit, School of Clinical Medicine, Institute of Metabolic Science, University of Cambridge, Cambridge, UK; 3 Precision Healthcare University Research Institute, Queen Mary University of London, London, UK; 4 Computational Medicine, Berlin Institute of Health at Charité-Universitätsmedizin Berlin, Berlin, Germany; 5 Human Genetics and Genomics, GSK Research and Development, Collegeville, PA USA; 6 Human Genetics and Genomics, GSK Research and Development, Cambridge, MA USA; 7 Institute of Health Informatics, University College London, London, UK; 8 National Institute for Health Research, Biomedical Research Centre, University College London Hospitals NHS Trust, London, UK; 9 Heidelberg Institute for Stem Cell Technology and Experimental Medicine, Heidelberg, Germany; 10 Division of Stem Cells and Cancer, Deutsches Krebsforschungszentrum (DKFZ) and DKFZ–ZMBH Alliance, Heidelberg, Germany; 11 Faculty of Biosciences, Heidelberg University, Heidelberg, Germany; 12 Genomic Sciences, Cellzome GmbH, GSK Research and Development, Heidelberg, Germany; 13 Berlin Institute of Health at Charité-Universitätsmedizin Berlin, Berlin, Germany; 14 Charité-Universitätsmedizin, Berlin, Germany; 15 Berlin Institute for Medical Systems Biology, Max Delbrück Center for Molecular Medicine in the Helmholtz Association, Berlin, Germany; 16 German Cancer Consortium (DKTK), Heidelberg, Germany; 17 Health Data Research UK, London, UK; 18 British Heart Foundation Data Science Centre, London, UK

**Keywords:** Predictive markers, Disease prevention, Risk factors

## Abstract

For many diseases there are delays in diagnosis due to a lack of objective biomarkers for disease onset. Here, in 41,931 individuals from the United Kingdom Biobank Pharma Proteomics Project, we integrated measurements of ~3,000 plasma proteins with clinical information to derive sparse prediction models for the 10-year incidence of 218 common and rare diseases (81–6,038 cases). We then compared prediction models developed using proteomic data with models developed using either basic clinical information alone or clinical information combined with data from 37 clinical assays. The predictive performance of sparse models including as few as 5 to 20 proteins was superior to the performance of models developed using basic clinical information for 67 pathologically diverse diseases (median delta C-index = 0.07; range = 0.02–0.31). Sparse protein models further outperformed models developed using basic information combined with clinical assay data for 52 diseases, including multiple myeloma, non-Hodgkin lymphoma, motor neuron disease, pulmonary fibrosis and dilated cardiomyopathy. For multiple myeloma, single-cell RNA sequencing from bone marrow in newly diagnosed patients showed that four of the five predictor proteins were expressed specifically in plasma cells, consistent with the strong predictive power of these proteins. External replication of sparse protein models in the EPIC-Norfolk study showed good generalizability for prediction of the six diseases tested. These findings show that sparse plasma protein signatures, including both disease-specific proteins and protein predictors shared across several diseases, offer clinically useful prediction of common and rare diseases.

## Abstract (web summary)

Proteomic prediction models developed using a large-scale dataset from the UK Biobank Pharma Proteomics Project were superior to clinical models for assessing the 10-year risk of 67 diseases across different types of pathology, including multiple myeloma, motor neuron disease, pulmonary fibrosis, celiac disease and dilated cardiomyopathy.

## Main

A central challenge in precision medicine is the development of clinically useful tools for identifying individuals at high risk, which may enable timely diagnosis, early initiation of treatment and improved patient outcomes^1^. Clinically recommended tools for predicting the risk of onset of diseases are used widely for heart attack and stroke (for example, the American College of Cardiology/American Heart Association 10-year risk equation)^2^ but for very few other diseases. Across diverse disease pathologies, diagnostic delays of months or years are reported from the initial onset of symptoms^3–5^. Over the last decades, single plasma proteins have become established as specific, diagnostic assays for a small number of diseases, including B-type natriuretic peptide (BNP) for heart failure, troponins for acute coronary syndromes and ubiquitin C-terminal hydrolase L1 (UCH-L1) and glial fibrillary acidic protein (GFAP) in traumatic brain injury^6^.

Broad capture plasma proteomics allows estimation of thousands of proteins and agnostic discovery studies not confined to a single disease of interest and represents a promising technology to accelerate progress towards this challenge. Plasma proteomic signatures capture health behaviors and current health status^7^, and may integrate the risk of ‘static’ genetic^8,9^ and dynamic environmental determinants of disease. Translatable, parsimonious models have been described. For example, a sparse protein signature, containing as few as three proteins, improved identification of a high-risk group for diabetes that is currently missed by screening strategies^10^.

Whether plasma proteomics may offer clinically useful predictive or mechanistic information across a wide range of diseases, alone or in combination, is unknown for several reasons. First, previous proteomic studies have had too few participants to evaluate rare and common diseases. Second, previous studies of disease onset have focused on a narrow set of common diseases^7,11–13^, rather than taking an agnostic discovery approach. Third, previous studies have not reported screening metrics compared with clinical models (without proteins), which may inform integration into health records and translational evaluation.

We used data from the United Kingdom (UK) Biobank Pharma Proteomics Project (UKB-PPP)—the largest proteomic experiment to date—to address the following objectives: (1) to systematically interrogate the 10-year predictive potential of the measurable plasma proteome across 218 pathologically diverse diseases, over and above models based on information obtained in usual care (without and with clinical assays) and polygenic risk scores; (2) to identify disease-specific protein predictors pointing to underlying etiological mechanisms, compared with those shared across diseases and (3) to determine whether the screening metrics of proteomic signatures for diseases meet, or exceed, those for blood assays used in current clinical practice.

## Results

**Fig. 1:**
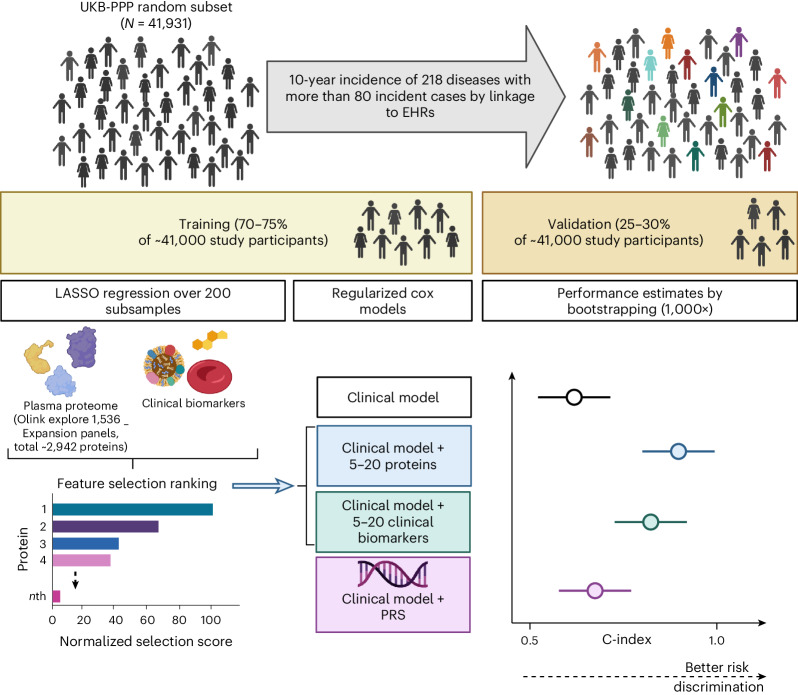
Study design. This cohort study is based on a random subset of UKB-PPP individuals (*N* = 41,931). The cohort was divided into training (including feature selection and optimization steps) and validation sets to develop sparse protein-based predictors (including 5–20 proteins from the Olink Explore 1536 and Explore Expansion panels) for 218 diseases defined using data from the UKB health-questionnaire, primary care, hospital episode statistics and cancer and death registries. Performance of models using protein signatures was compared with models using basic clinical information alone or using basic clinical information combined with clinical assay data or genome-wide PGS. Created with BioRender.com.

**Extended Data Fig. 1:**
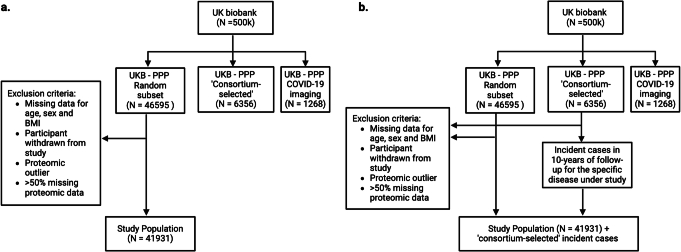
Overview of the study design in the context of the UK biobank Pharma Proteomics Project (UKB-PPP). **a**, Study design used for 193 diseases for which only participants from the randomly selected subset were included in the analysis. **b**, Study design used for 25 less common diseases were incident cases within 10 years of follow-up for the specific disease under study were included in the analysis. Created with BioRender.com.

We carried out a cohort study in the UKB-PPP, where plasma proteomic profiling was done with the Olink Explore 1536 and Explore Expansion platform, targeting 2,923 unique proteins by 2,941 assays. We developed prediction models for 218 diseases, with more than 80 incident cases within 10 years of follow-up in the random subset of the UKB-PPP (*N* = 41,931; 193 diseases) (Fig. 1), or by including incident cases within the ‘consortium-selected’ subset (25 diseases out of the 218) (Supplementary Tables 1 and 2 and Extended Data Fig. 1). Disease definitions were based on validated phenotypes previously described^14^ by integrating data from primary care (available for only a subset of individuals), hospital episode statistics, cancer and death registries and from UKB health questionnaires including self-reported illnesses. We excluded prevalent cases (first occurrence before or up to the baseline assessment visit) or incident cases recorded within the first 6 months of follow-up (Methods).

## Sparse protein signatures improved prediction over clinical models

Clinical models, including age, sex, body mass index (BMI), self-reported ethnicity, smoking status, alcohol consumption and self-reported paternal or maternal history for 15 diseases for which this was assessed at baseline, showed a median concordance index (C-index) = 0.64 (interquartile range (IQR) = 0.58–0.72), with highest performance achieved for endocrine and cardiovascular diseases. For 163 diseases, five proteins alone—not considering any other information—performed as well as the clinical model, and significantly better for an additional 30 diseases (Supplementary Fig. 1 and Supplementary Table 3).

**Fig. 2:**
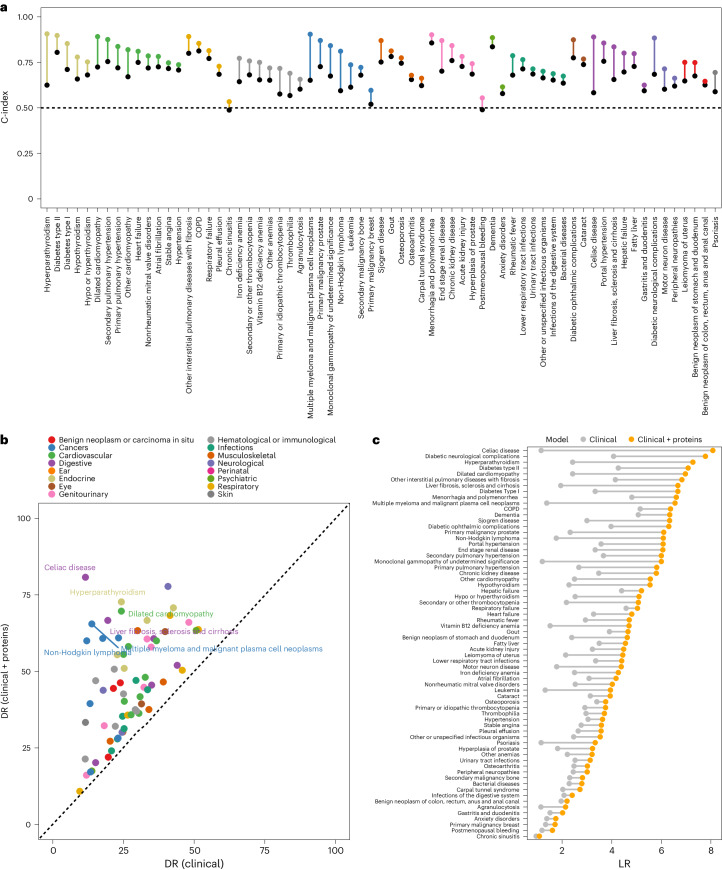
Improvement in predictive performance of disease incidence by addition of proteomic information on top of basic clinical risk factors for 67 diseases. **a**, Improvement in C-index by the addition of signatures comprising 5–20 proteins (coloured dots) over the benchmark clinical model (black dots). **b**, Comparison of DRs (at a 10% FPR) achieved by protein-based and clinical models. **c**, Improvement in LRs by the addition of signatures comprising 5–20 proteins (orange) over the benchmark clinical model (gray).

**Fig. 3:**
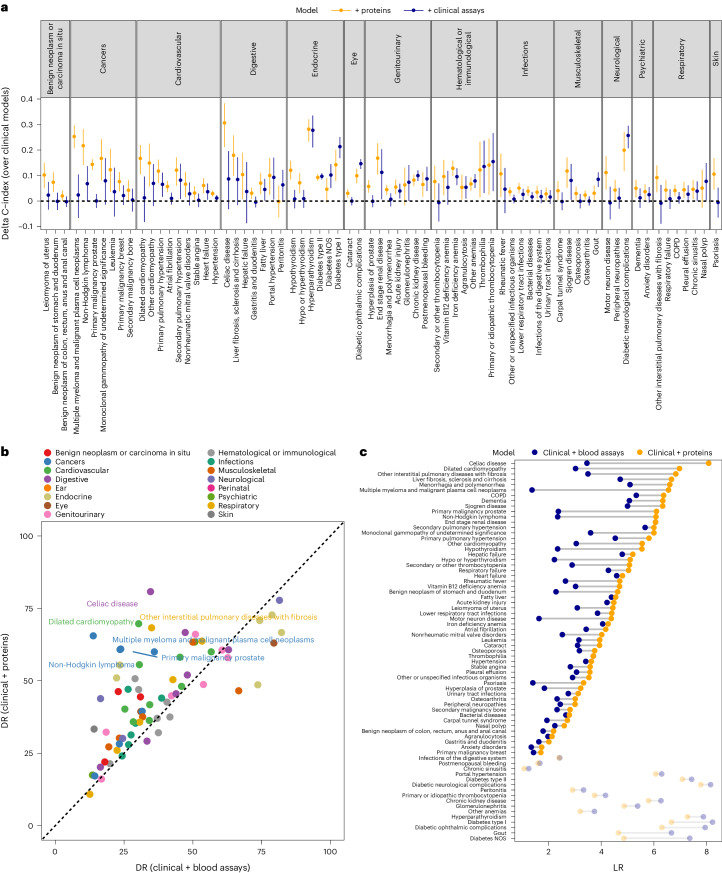
Comparison of predictive performance between protein-based (clinical risk factors + proteins) and biomarker-based (clinical risk factors + blood assays) models. **a**, Comparison of C-index by the addition of protein-based (orange) or biomarker-based models (blue) onto clinical risk factors. We only show those diseases for which the C-index was improved significantly by addition of either proteins or clinical assays onto the clinical risk factors. We present the mean C-index and the 95% CI. **b**, Comparison of DRs (at a 10% FPR) achieved by protein-based and biomarker-based models. **c**, Comparison of LRs for protein-based (orange) or biomarker-based models (gray).

**Extended Data Fig. 2:**
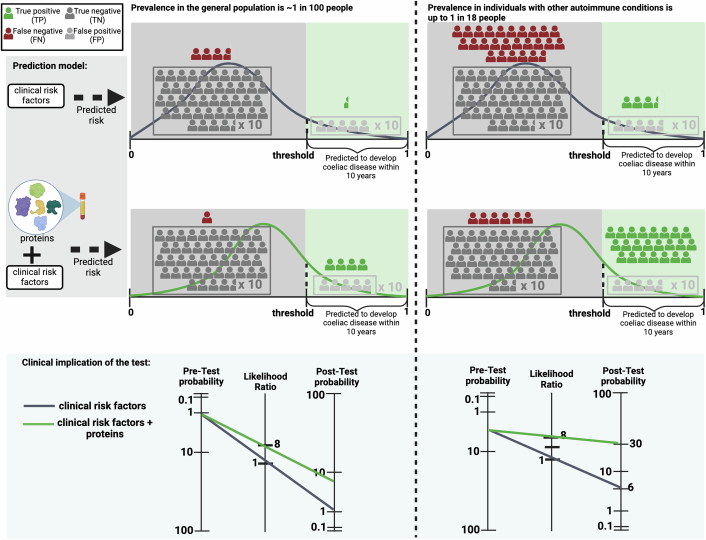
Example of the improvement from proteomically informed screening strategies for coeliac disease. We present two scenarios, in which screening is performed in 1) the general population and 2) a high-risk population (individuals with other autoimmune conditions). According to their predicted risk, individuals are classified as ‘positive’ (those predicted to develop coeliac disease within the next 10 years) or ‘negatives’ (not predicted at risk of coeliac disease). We illustrate the number of true positives, false positives, true negative and false negative that would be obtained according to the detection rate we estimated for coeliac disease in UK biobank at a 10% false positive rate. We further represent the pre-test probability, likelihood ratio (LR) and post-test probability in the two different scenarios (general population and high-risk population). Created with BioRender.com.

For 67 rare and common diseases, addition of 5 to 20 proteins significantly improved clinical models (median increase in C-index = 0.07, range = 0.02–0.31) (Fig. 2a and Supplementary Table 4). Diseases for which proteins improved clinical models (95% confidence intervals (CI) of improvement in C-index (delta C-index) > 0) included multiple myeloma (MM) (delta C-index = 0.25 (95% CI 0.20–0.29, likelihood ratio (LR) = 6.55), non-Hodgkin lymphoma (delta C-index = 0.21 (0.14–0.28), LR = 6.08), pulmonary fibrosis (delta C-index = 0.09 (0.03–0.14), LR = 6.83), celiac disease (delta C-index = 0.31 (0.21–0.38), LR = 8.07), dilated cardiomyopathy (delta C-index = 0.17 (0.10–0.22), LR = 6.97) and motor neuron disease (delta C-index = 0.11 (95% CI 0.04–0.16), LR = 4.38) (Fig. 2a). Across these 67 diseases, the median detection rate (at a 10% false positive rate (FPR), detection rate (DR)_10_) was 45.5% (range 10.8–80.8%), compared with 25% (range 9.5–51.2%) for the clinical model (Fig. 2b and Supplementary Table 5). The median LR was 4.55 (range 1.08–8.07) for these 67 diseases, representing improvements ranging from 0.12 to 6.92 over the clinical models (Fig. 2c). For example, applying a protein-informed test for celiac disease (LR = 8.08) would result in detecting 80.8% of cases, while retaining an acceptable proportion of 10% false positives (Extended Data Fig. 2). The mean category-free net reclassification improvement across these was 0.10 (25th–75th percentile = 0.03–0.15; Supplementary Table 6), and mean integrated discrimination improvement 4.79% (25th–75th percentile = 1.7–6.4%; Supplementary Table 7). Models additionally including blood assay results (Supplementary Table 8) showed significantly improved prediction over clinical models for only 28 diseases (median delta C-index = 0.08, range = 0.01–0.28) (Fig. 3 and Supplementary Table 9). For 52 of the 67 diseases, protein-based models achieved higher LRs (range 0.13–5.17) in comparison with clinical models with blood assays (Fig. 3b,c and Supplementary Table 10). To accelerate the use and translational potential of our findings, we generated an open-access interactive web resource that enables the scientific community to easily visualize post-test probabilities^15^ based on derived LRs across all tested diseases (https://omicscience.org/apps/protpred).

**Extended Data Fig. 3:**
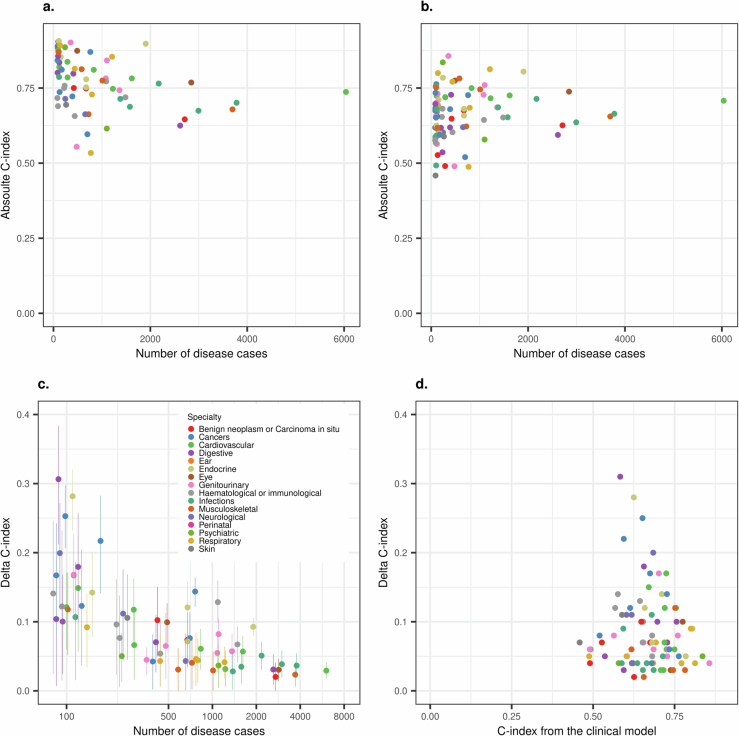
Predictive performance is not related with the number of incident cases. **a**, Predictive performance (C-index) of protein-based models, across 67 diseases for which these outperformed clinical models, was not correlated with the number of incident cases within 10 years of follow-up. **b**, Predictive performance (C-index) of the clinical models was not correlated with the number of incident cases within 10 years of follow-up. **c**, Improvement in predictive performance (delta C-index) of protein-based models over clinical models appeared to be the largest for diseases less frequent among the UKB population. We present the mean C-index with a 95% confidence interval shown by the error bars. **d**, Improvement in predictive performance (delta C-index) of protein-based models was not correlated with baseline prediction of the clinical models.

Compared with the single most informative protein, sparse protein signatures (5–20 proteins) had an average 5.4% improvement in C-index over clinical models, across diseases that achieved significant improvements. For 64% of these, performance saturation was achieved by including a maximum of five to ten proteins. Among the 67 diseases with significantly improved prediction by proteins, there was a more than eightfold enrichment for hematological or immunological diseases (odds ratio = 8.6; *P* = 0.004). Prediction models were on average improved more (by proteins) for less common diseases (Pearson *r* between *N* incident cases and change in C-index = −0.51; *P* value = 9.3 × 10^−3^) (Extended Data Fig. 3). However, this correlation was not evident across all 218 diseases tested (Pearson *r* = −0.04, *P* value = 0.52) and downsampling of incident cases (for hypertension, for example) did not result in inflation of improvements in C-index (Supplementary Table 11). Selected proteins for the 67 improved diseases showed little evidence of being specifically enriched or under-represented among Olink panels, with the exception of the cardiometabolic panel (fold change, 1.58; *P* value = 0.001) and the oncology II panel (fold change, 0.64; *P* value = 0.007). A total of 19 of the 67 diseases showed enrichment for tissue-specific proteins (for example, lymphoid tissue for MM) or certain pathways, but only a few of these seemed directly related to known disease pathology, such as cholesterol metabolism being enriched among proteins predicting stable angina (fold change, 27.0; *Q* value = 2.4 × 10^−4^).

**Extended Data Fig. 4:**
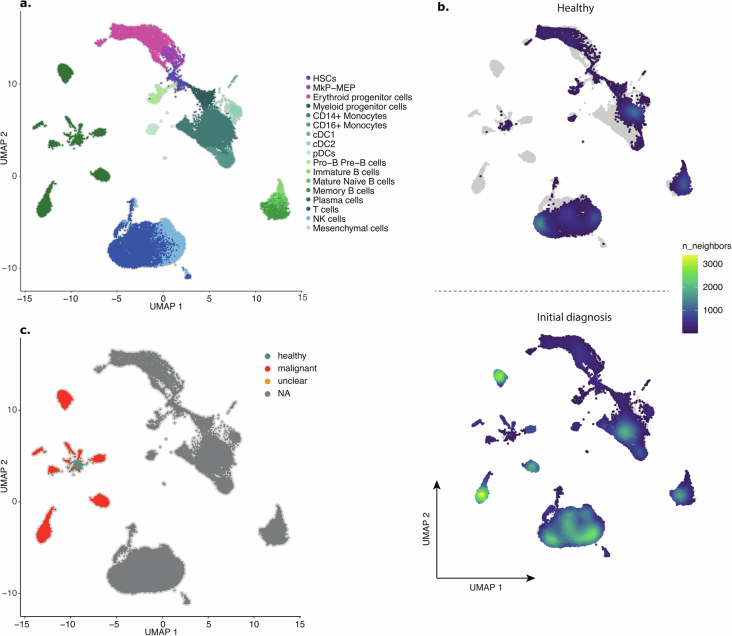
Bone marrow (BM) immune microenvironment of multiple myeloma (MM) patients captured by scRNA-seq. **a**, UMAP representation of scRNA-seq data of 11 BM samples from MM patients at initial diagnosis and 3 healthy controls. Cell types are highlighted by color. **b**, UMAP from (A) split by clinical state (healthy, initial diagnosis). Cell density and distribution is illustrated by color. **c**, BM UMAP from (A) highlighting the plasma cell state healthy (green), malignant (red) and unclear (yellow) based on copy number aberrations detected by inferCNV.

**Extended Data Fig. 5:**
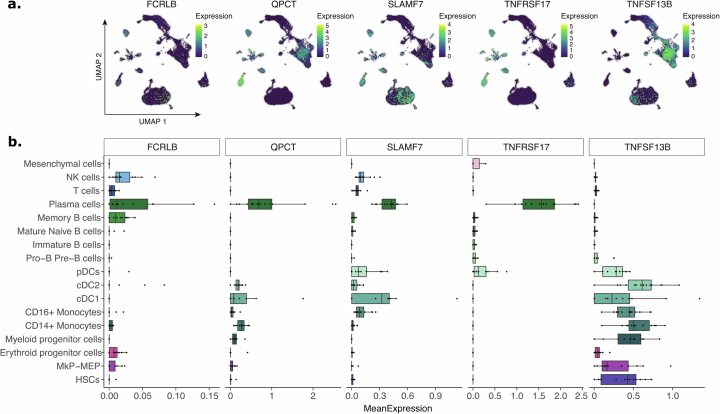
Gene expression levels of predictor proteins within the bone marrow (BM) immune ecosystem. **a**, UMAP with highlighted gene expression of predictor proteins across all celltypes in the BM. **b**, Mean gene expression levels of predictor proteins within the BM split by cell type. Data are presented as median values; box edges are 1st and 3rd quartiles; and whiskers represent 1.5× interquartile range (N = 3 - 14).

**Extended Data Fig. 6:**
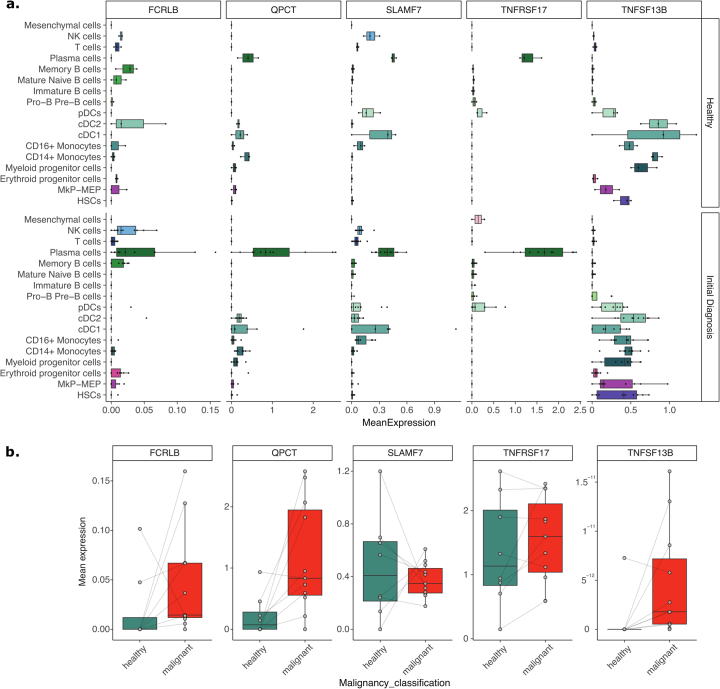
Gene expression levels of predictor proteins between healthy and malignant state and cells in the bone marrow immune environment of multiple myeloma (MM) patients. **a**, Mean gene expression levels of predictor proteins within the BM split by cell type and clinical state (healthy, initital diagnosis). **b**, Box plots illustrating mean gene expression of predictor proteins within healthy versus malignant plasma cells of MM patients at initial diagnosis as characterized by inferCNV. Data are presented as median values; box edges are 1st and 3rd quartiles; and whiskers represent 1.5× interquartile range (N healthy = 8, N malignant = 11).

For MM, we were able to integrate single-cell RNA sequencing (scRNA-seq) data of the bone marrow (BM) immune microenvironment of 11 newly diagnosed MM patients and three healthy controls^16^ (Extended Data Fig. 4). Across 17 different BM cell types, we found that four (FCRLB, QPCT, SLAMF7 and TNFRSF17) of the five identified predictor proteins were expressed most abundantly in plasma cells (Extended Data Fig. 5 and Supplementary Table 12), suggesting these proteins may act as markers of plasma cell levels, which are elevated at primordial stages of MM development. Malignancy classification of BM plasma cells in the same dataset (Extended Data Fig. 4c), based on detected copy number aberrations using inferCNV^17^, showed that upregulation of *FCRLB* and *QPCT* expression in plasma cells from MM patients was driven by malignant plasma cells (Extended Data Fig. 6 and Supplementary Table 13). We also observed slight upregulation of *TNFSF13B* expression in malignant plasma cells but, because of the nonspecific gene expression profile of *TNFSF13B* in BM, this increase contributed only minimally to its overall expression.

For 14 of 41 diseases tested (from the 67 that improved by proteins that had enough cases for stratified analyses; Methods), predictive performance differed significantly between men and women. For 28 additional diseases, significant improvements in prediction by proteins were identified only in sex-stratified analyses (Supplementary Fig. 2a and Supplementary Table 14). For all other 79 diseases, performance was found to be similar between men and women (Pearson *r* between C-indices = 0.92, *P* value = <2.2 × 10^−16^) (Methods). In age-of-onset-stratified analyses (<65 versus ≥65 years at onset), performance differed significantly for 39 of the 47 diseases tested, from the 67 that improved by proteins with enough cases (Methods). Predictive performance was improved by proteins for another 75 diseases in age-of-onset-stratified analyses only. For all other 20 diseases, performance was similar between younger and older disease onset (Pearson *r* = 0.94, *P* value = 3.85 × 10^−10^) (Supplementary Fig. 2b and Supplementary Table 15).

**Extended Data Fig. 7:**
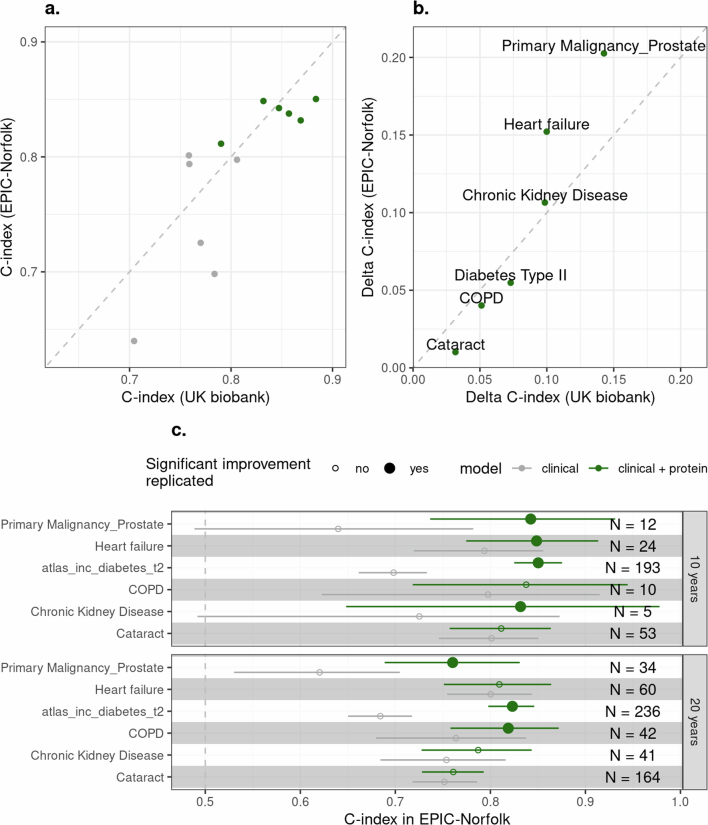
External validation in the EPIC-Norfolk study. **a**, Comparison of C-index achieved by UKB-trained models in the UKB validation set and in EPIC-Norfolk (for 10-year incidence). **b**, Comparison of the improvement in C-index of the protein-based models over the clinical model in UKB and in EPIC-Norfolk (for 10-year incidence). **c**, Replication of the improvement provided by protein signatures identified in UKB, over clinical models, in the EPIC-Norfolk study. Predictive performance for 10- and 20-year incidence are shown. We present the median C-index with a 95% confidence interval N: Number of incident disease cases.

Although the breadth of our study and the scale and novelty of the UKB-PPP data did not enable external replication for most protein models, we were able to assess generalizability of results for 6 of the 67 diseases for which proteins improved prediction over and above clinical models in the European Prospective Investigation into Cancer (EPIC)-Norfolk study (*N* = 295–1,116; *N* incident cases = 5–236; Supplementary Tables 16 and 17; Methods). Models trained using the UKB-PPP data achieved highly comparable C-indexes (Pearson *r* = 0.81; *P* value = 0.002; Extended Data Fig. 7a) and improvements in prediction by the proteins informed models over the clinical models (Pearson *r* = 0.97; *P* value = 0.001; Extended Data Fig. 7b) in the EPIC-Norfolk study. This indicates generalizability of the predictive proteins and models trained in UKB. While models trained in UKB were not explicitly trained for prediction of more than 10-year incidence, UKB-trained models retained substantial performance for prediction of 20-year incidence in EPIC-Norfolk over and above clinical models (Extended Data Fig. 7c). We further replicated significant improvements in predictive performance achieved by protein signatures over the clinical benchmarks for five of the six diseases tested (Extended Data Fig. 7c). For one of these diseases, chronic obstructive pulmonary disease (COPD), we were only able to replicate the improvement by testing prediction of 20-year incidence, most likely due to few incident cases within 10 years of follow-up.

## Proteins predicting several diseases

**Fig. 4:**
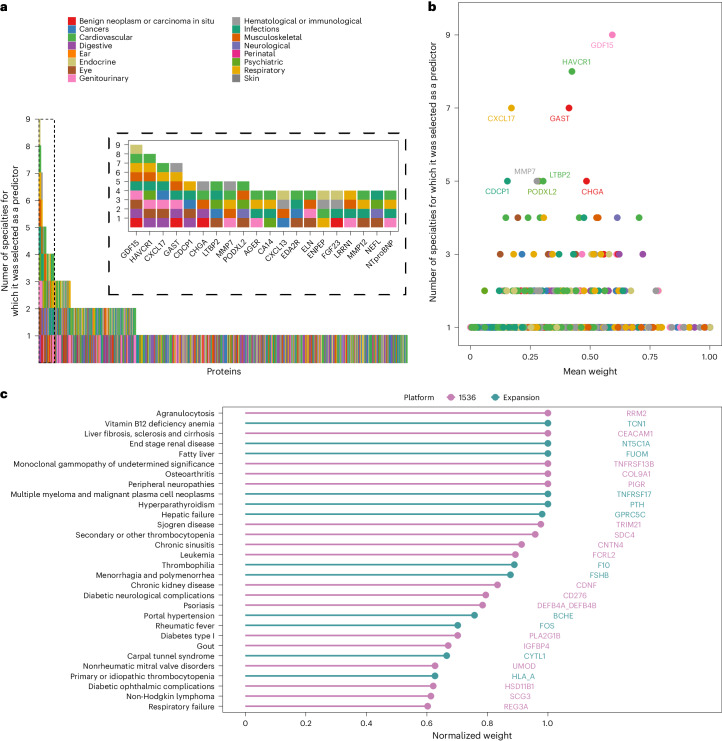
Disease specificity of predictor proteins. **a**, Number of disease specialties for which a protein was selected as a predictor across the 67 diseases for which the C-index was significantly improved by a protein signature as compared with the clinical model. The box with the dashed lines provide a zoomed version of the plot for proteins that were selected across four or more clinical specialties. **b**, Mean model weights for each protein (normalized to the top predictor) across diseases for which it was selected as a predictor (out of the 67 improved diseases). **c**, Disease-specific proteins are shown as those selected for only one disease with a normalized weight >0.6. Platform: Protein included in the Olink Explore 1536 panels or the Olink Explore Expansion panels.

**Extended Data Fig. 8:**
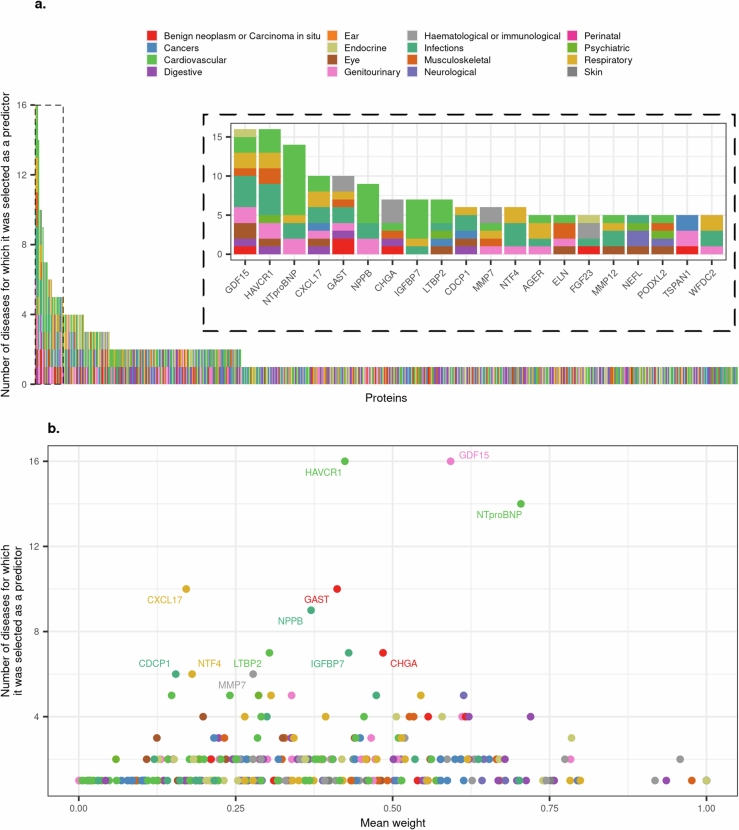
Disease specificity of predictor proteins. **a**, Number of individuals diseases for which a protein was selected as a predictor across the 67 diseases. These were diseases for which the C-index was significantly improved or improved by more than 0.4 over the clinical model. **b**, Average contribution of proteins across diseases. Average weights (normalised to the top predictor) from the optimised prediction models for each protein (across diseases for which it was selected as a predictor).

**Extended Data Fig. 9:**
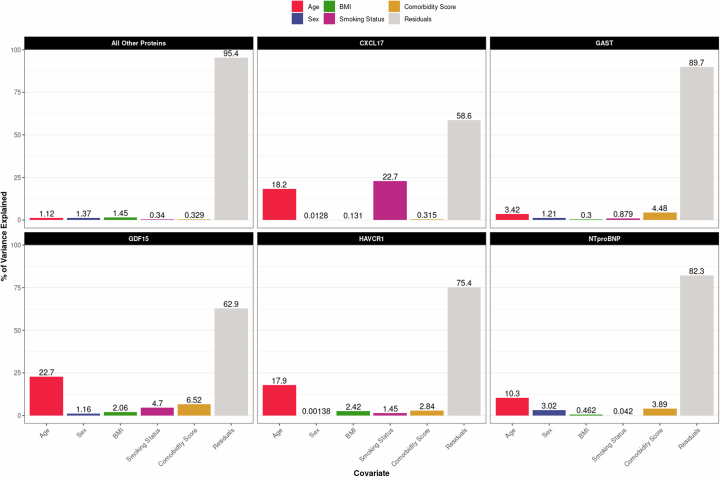
Proportion of variance explained in plasma levels of proteins predictive across more than 10 diseases by demographic characteristics. Proportion of variance by age, sex, body mass index (BMI), smoking status and a comorbidity score (see Methods) in a joint model. This is compared the average variance explained by each of these characteristics in plasma levels of all other proteins.

The 67 prediction models with clinically relevant improvements, included a total of 501 protein targets, of which 147 were selected for two or more (range 2–16) diseases (Extended Data Fig. 8), most (~89%) of which were selected across two or more clinical specialties (range 2–9) (Fig. 4a). On average, these had a relatively lower contribution for prediction of individual diseases, in comparison with highly specific proteins (Fig. 4b), and we further observed no enrichment of specific biological pathways. Age was the main correlate of four out of the five proteins that were predictive across more than ten diseases, and smoking status was the main correlate for CXCL17 (Extended Data Fig. 9), but these proteins still provided improvements in prediction over and above these conventional risk factors.

## Proteins specifically predicting one disease

We identified proteins solely and strongly predictive for only one disease (Fig. 4c and Supplementary Table 18). Feature selection scores for these proteins across other diseases were, on average, 86% lower compared with the selection score for the specific disease (Supplementary Fig. 3). These proteins included TNF receptor superfamily member 17 (TNFRSF17 or B cell maturation antigen)—a specific predictor for MM—and TNFRSF13B—a strong predictor of monoclonal gammopathy of undetermined significance (MGUS), a condition that precedes the development of MM (at a rate of ~1 in 100 MGUS cases developing MM per year^18^). Here, we provide evidence that increased plasma levels of these receptors (Supplementary Table 19) are strongly predictive of future onset for these blood cancers. Previous studies have already suggested an association between plasma TNFRSF17 and progression from MGUS to MM^19^. Here we identified the added value of a five-protein protein signature, which improved discrimination by 7% over clinical risk factors + TNFRSF17 alone.

## Polygenic risk scores compared with clinical models and protein models

**Extended Data Fig. 10:**
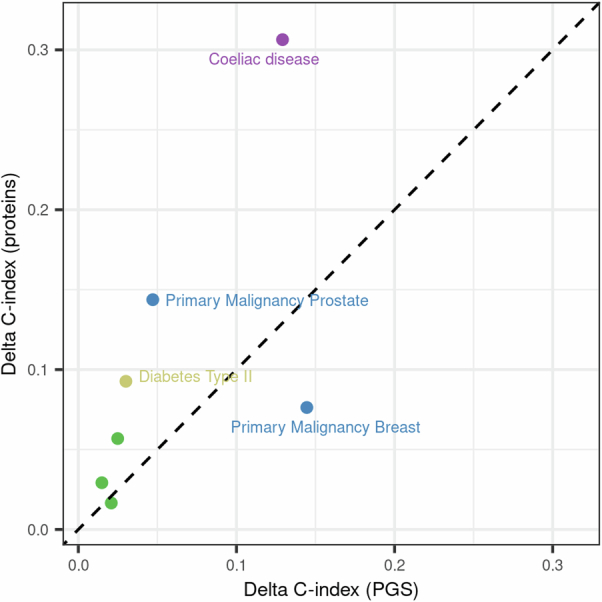
Comparison of the predictive performance of proteins and PGS over clinical models. Comparison of the improvement in predictive performance over clinical models (delta C-index) provided by PGS and 5–20 proteins. Only 7 diseases for which the PGS provided a significant improvement in performance are shown.

For 23 diseases for which polygenic risk scores (PGS) were available in UKB, we found that PGS improved prediction significantly over clinical models (without blood assays) for only seven diseases, but with clinically negligible improvements (median delta C-index = 0.03, range = 0.01–0.14) (Supplementary Table 20) compared with those provided by proteins for those seven diseases (median delta C-index = 0.08, range = 0.02–0.30). Proteins outperformed PGS for all of these diseases, except for breast cancer (Extended Data Fig. 10).

## Screening metrics for protein and clinical models

**Fig. 5:**
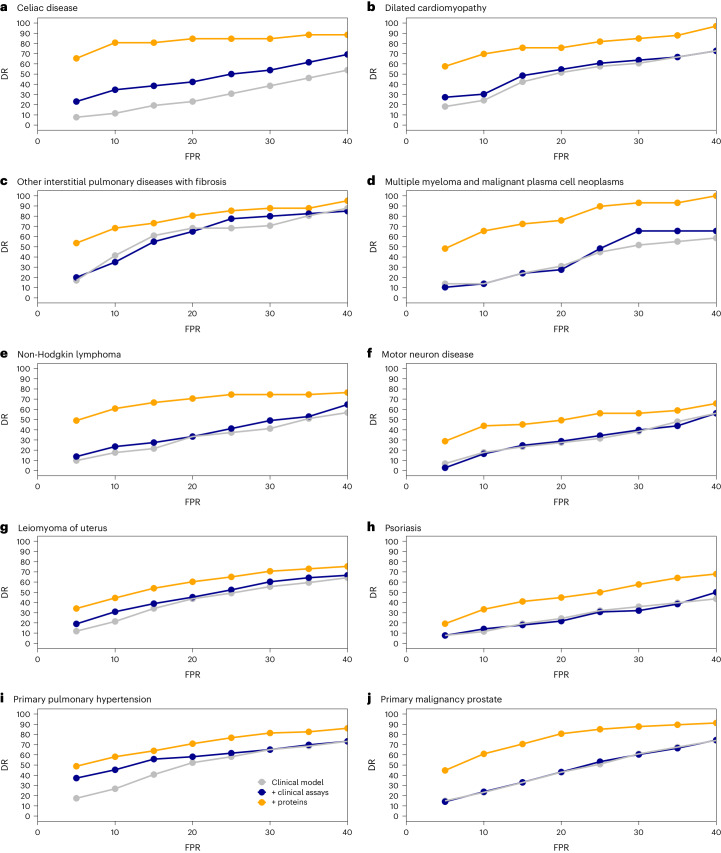
DR curves. DRs across different FPR thresholds for selected disease examples, which were identified as those most likely to benefit from proteomic prediction over clinical risk factors, clinical assays and PGS. **a**, Celiac disease (protein signature: TGM2, NOS2, ITGB7, CD160, PPP1R14D, RBP2, CCL25, MLN, FGF19, HMOX1, CEND1, MILR1, CDH2, CKMT1A_CKMT1B, CPA2, GTF2IRD1, SEPTIN3, BCL2L15, FABP2, HSD17B14). **b**, Dilated cardiomyopathy (protein signature: HRC, TNNI3, TPBGL, NPPB, NTproBNP). **c**, Other interstitial pulmonary disease with fibrosis (protein signature: CA4, CEACAM6, GDF15, SFTPD and WFDC2); **d**, MM and malignant cell neoplasms (protein signature: FCRLB, QPCT, SLAMF7, TNFRSF17, TNFSF13B); **e**, non-Hodgkin lymphoma (protein signature: BCL2, CXCL13, IL10, PDCD1, SCG3); **f**, motor neuron disease (protein signature: CST5, EGFLAM, NEFL, PODXL2 and TMED10); **g**, leiomyoma of uterus (protein signature: BMP4, CDH3, CHRDL2, DNPEP, FGF23, GFRAL, LEFTY2, PAEP, SEZ6L2, TSPAN1); **h**, psoriasis (protein signature: DEFB4A_DEFB4B, IL19, KCTD5, PI3, PRKD2); **i**, primary pulmonary hypertension (protein signature: NPPB, NTproBNP, ROBO2, ENPEP, FGFBP2, LTBP2, SFRP1, ACP5, SPON1, CA4, SLC34A3, ACE2, AHSG, SERPINA7, SLC44A4, CDC123, SPINK8, LYPLA2, S100A3, MFAP4); **j**, primary malignancy prostate (protein signature: ADAMTS15, IL17A, INSL3, KLK3, LECT2, LTBP2, PRR5, SCARF2, SPINT3, TSPAN1).

We observed consistently superior screening metrics across all conditions for a wide range of FPRs (5–40%; Fig. 5). At a 20% FPR, proteomic prediction identified individuals at high risk for pulmonary fibrosis (including CA4, CEACAM6, GDF15, SFTPD and WFDC2; DR = 80%) and dilated cardiomyopathy (including HRC, TNNI3, TPBGL, NPPB and NTproBNP; DR = 75%). At a low FPR (5%), proteomic prediction identified individuals at high risk for MM (FCRLB, QPCT, SLAMF7, TNFRSF17 and TNFSF13B; DR = 50%), non-Hodgkin lymphoma (BCL2, CXCL13, IL10, PDCD1 and SCG3; DR = 55%) and motor neuron disease (including CST5, EGFLAM, NEFL, PODXL2 and TMED10; DR = 29%).

## Sensitivity analyses

In sensitivity analyses, we found that adding a larger set of proteins included in Olink’s Explore Expansion panels (Methods) did not generally improve model performance compared with the first release of 1,463 proteins (Supplementary Fig. 4 and Supplementary Table 4). However, improvements for selected diseases were obtained by including a specific predictive biomarker (captured only in the Expansion panels), such as TCN1 (a vitamin B12 binding protein) for vitamin B12 deficiency anemia, KLK3 (prostate-specific antigen) for prostate cancer or, F10 (a coagulation factor that converts prothrombin into thrombin) and PROS1 (an anticoagulant protein) for thrombophilia (Supplementary Fig. 4). Protein-based models trained on 10-year incidence performed equally well when restricting the follow-up time to 5 years (Pearson *r* = 0.96; Supplementary Fig. 5a), although clinical models appeared to have systematically lower performances indices up to 5 years (Pearson *r* = 0.88; Supplementary Fig. 5b).

## Discussion

We demonstrate the potential of sparse protein signatures to improve the prediction of disease onset across common and rare diseases. By integrating ~3,000 broad-capture plasma proteins with electronic health records (EHRs), we showed that for 52 of 218 diseases studied, adding proteins was the single best prediction model, not only superior to commonly used patient characteristics, but also to a large array of blood assays in clinical use and PGS (where available). For many diseases, broad-capture proteomic technologies offer new possibilities to address delays in diagnosis, the first blood-based biomarkers and the first evidence of better prediction models compared with current practice (Supplementary Table 21). Our results highlight where plasma proteomic signatures may inform the need for, and design of, therapeutic clinical trials.

The wide spectrum of diseases that we studied enabled discovery of disease-proteomic signatures with the strongest screening metrics. The proteomic signatures that we report have screening metrics that were comparable with, or exceeded, those of blood tests currently used as diagnostic tests (for other diseases). Previous studies in a small number of diseases have investigated the predictive^7,11–13^ or prognostic^20^ potential of the circulating proteome. We found that for almost two-thirds (61%) of the superior protein models, a positive test, that is, a predicted risk above the risk cut-off, translated into a fourfold increased risk of developing the disease compared with a negative one. Specifically, for 14 diseases, the LR achieved by protein-based models was higher than for a signature including prostate-specific antigen (KLK3) for prostate cancer, which is used in currently implemented screening programs^21^. Sparse protein signatures (5–20 proteins) offer the opportunity to assess a limited set of proteins at a cost much below a broad-capture discovery proteomic assay. The fact that we identified strong predictive signatures in the nonfasting UKB samples further suggested feasibility of measurement in clinical practice. Our development of ‘sparse’ signatures was designed to facilitate translation of findings, which will require absolute quantification of proteins by clinical grade assays, something that is more feasible and affordable for small panels or numbers of proteins. Furthermore, our extremely sparse signatures performed better or equally for most of the 22 diseases for which complex deep learning models had been developed, in the same UKB-PPP study, including 1,536 proteins (Olink Explore 1536) and 54 clinical variables (including demographic, lifestyle, physical measures, medical and family history and blood clinical assays)^22^ (Supplementary Table 22). This demonstrates the advantage and robustness of our approach.

We identified specific and strongly predictive proteins, pointing to underlying pathways conferring disease risk. Here, we show that up to 10 years before diagnosis, higher plasma levels of TNFRSF17 and TNFRSF13B, receptors for BAFF and APRIL, were strong, specific predictors of increased risk of MM and MGUS, respectively. These signaling pathways have been shown to promote MM growth^23,24^. In turn, decreased plasma TNFSF13B, was further shown to be predictive of higher risk for MM. Anti-TNFRSF17 agents, including antibody–drug conjugates, T cell engagers bispecific antibodies and cellular therapy with chimeric antigen receptor T cells, are approved for the treatment of refractory MM^25–29^. Clinical trials exploring earlier implementation have started providing evidence for the safety and effectiveness of anti-TNFRSF17 agents in early lines of treatment^30^. Our results demonstrated the potential for implementation of proteomic screening, in a preventative manner even years before the onset of overt MM, to identify the subgroup of individuals at highest risk, and highlight the possibility to test whether they represent those who would eventually benefit the most from assessment of anti-TNFRSF17 as earlier lines of treatment. Pulmonary fibrosis may be delayed due to misdiagnosis of other common respiratory or cardiovascular diseases^31^. The proteomic signature should be evaluated to identify who might benefit from enhanced surveillance through lung function tests and lung imaging, potentially enabling early treatment to maximize preservation of lung function, now possible with anti-fibrotic therapies^32^. For dilated cardiomyopathy, proteomic signatures could be evaluated for their potential to inform electrocardiogram and echo surveillance in people without a known genetic cause (up to 60% of cases^33,34^).

We found proteins predictive across several diseases and clinical specialties, consistent with shared etiologies, including adaptations to ageing. Gastrin, for example, is well known for its role in production of hydrochloric acid, gastric motility and associations with gastrointestinal cancers and digestive system diseases^35^. However, our results highlighted associations with a wider range of diseases, including vitamin deficiencies, osteoporosis, infections and acute kidney injury. Associations of proteins with ‘acute’ conditions such as infections might point to underlying susceptibility to an event through mechanisms that may point to impaired immune response or generalized frailty among others. Proof-of-principle studies have suggested that a single ‘omics’ signature may predict risk of onset across several diseases at once^36^. Although our results point to some proteins as possible markers of multimorbidity, the potential for leveraging pleiotropic proteins to develop a customized, small signature for prediction across several diseases remains to be explored.

We observed evidence that superior model performance using proteins was achieved more often for rarer diseases and diseases for which blood is an important compartment, such as hematological cancers, as discussed for MM. While the pathological connections of the blood plasma proteome to the latter categories of diseases is intriguing, the stronger improvement among rarer conditions might be explained by less phenotypic and molecular heterogeneity compared with common complex disorders like heart failure or type 2 diabetes (T2D). However, we currently lack systematic data-driven information on phenotypic risk factors for rare diseases. Future work should focus on exploring the improvement of protein biomarkers over systematically identified clinical risk factors for rarer conditions.

Substantial efforts have been made to improve genome-wide PGS and have led to arguments in favor of their potential utility for identification of individuals at high risk of disease onset^8,9,37^. However, our results highlighted their poor performance, compared with what can be achieved by up to 20 proteins only, in contrast to the information on millions of variants which are incorporated by PGS. This might be best explained by the dynamic nature of circulating protein signatures, which may in turn reflect changes in risk in response to environmental exposures^38^, as opposed to the ‘static’ nature of PGS. Future work might explore how proteomics compares with additional omics layers of information for prediction of future disease risk.

Our study has important limitations. First, our results require validation in external studies, in ethnically diverse populations and in cohorts with differing pre-test probabilities of disease (UKB has a healthy participant effect^39^). Second, although we report the largest proteomic experiment to date, larger sample sizes are required to estimate detection rates for rarer diseases, and over shorter clinically relevant time frames (for example, 1–5 years), depending on the underlying specific disease etiology. Third, evaluations against clinical diagnostic markers not available in UKB are required, including M-protein for MM, and IgA/IgG antibodies and anti-transglutaminase for celiac disease. Further, selected protein candidates might be early indicators of asymptomatic or dormant diseases processes that otherwise are associated with a significant delay in the diagnosis and recording in EHRs. Fourth, clinical translation will require development and validation of absolute quantification protein assays as opposed to the relative quantification provided by current proteomic platforms. We also note that the preselection of proteins on the Olink Explore platform, as any targeted assay, restricts the discovery space of new biomarker candidates upfront and that emerging untargeted mass spectrometry-based assays will probably reveal additional markers. Finally, we observed evidence that plasma proteins are superior in the prediction of diseases belonging to certain clinical specialties, whereas other diseases, for example, infectious or highly compartmentalized (for example, eye diseases), will require other types of tissue samples or entirely different clinical information to be better predicted.

In conclusion, we demonstrate that sparse plasma protein signatures when integrated with EHRs may offer new, improved prediction over standard clinical assays for common and rare diseases, through disease-specific proteins and protein predictors shared across several diseases.

## Methods

### Study design

The UKB study is a population-based cohort of around half a million participants from the UK aged between 40 and 59 years who were recruited between 2006 and 2010 (baseline assessment). Deep phenotype and genetic data are available for participants, including blood and urine biomarkers, whole-body imaging, lifestyle indicators, physical and anthropometric measurements, genome-wide genotyping, exome and genome sequencing. Follow-up is currently ongoing, and participants are further linked to routinely collected EHRs. Detailed information is available at https://biobank.ndph.ox.ac.uk/showcase/.

Proteomic profiling was performed in EDTA-plasma samples from ~54,000 UKB participants as part of the UKB-PPP. Details of the sample selection and sample handling have been described previously^40^. Briefly, the study design included three elements: (1) a randomized subset of 46,595 individuals; (2) 6,356 individuals selected by the UKB-PPP consortium members (‘consortium selected’), in which proteomic profiling was done on samples from the baseline assessment and (3) 1,268 individuals who participated in a COVID-19 imaging study with repeated imaging at several visits.

We carried out a cohort study in the UKB-PPP to develop, validate and compare predictive models with and without proteins. While the randomized subset was representative of the entire UKB population, ‘consortium selected’ participants had different baseline characteristics for common risk factors (on average older, higher BMI and more smokers) and were enriched in cases for 122 different diseases^40^. Therefore, we based analyses on individuals from the randomized subset excluding those with missing data for age, sex and BMI, or who failed quality control (QC) criteria for proteomic measurements (*N* = 41,931). For 25 less frequent diseases we further included incident cases occurring within the ‘consortium-selected’ participants (Supplementary Table 1). UKB has approval from the North West Multi-Centre Research Ethics Committee as a Research tissue biobank (REC reference 11/NW/0382). Participants provided written informed consent.

### Clinical risk information

Clinical risk information (without blood assays) recommended as part of usual primary care, was obtained from UKB health questionnaires. This included: age at baseline, self-reported ethnicity, smoking status, alcohol consumption, paternal or maternal history for 15 individual diseases available (datafield IDs 20197 and 20110; Supplementary Table 1), and measured BMI. We further included 37 of the most widely performed blood assays (16 of these are based on proteins), which were assessed in all UKB participants. These included 28 blood assays (UKB Category 17518) and 9 blood cell traits (UKB Category 100081) (leukocyte, lymphocyte, monocyte, neutrophil, eosinophil, basophil, platelet count, hemoglobin concentration and hematocrit percentage), and refer to these 37 blood-based tests^41^ (Supplementary Table 8) as clinical assays. Estrogen and rheumatoid factor were not included in the analyses given these had more than 50% of missing values. For the *n* = 9 blood cell traits, we excluded blood cell measures from individuals with extreme values or relevant medical conditions as described previously^42^. Relevant medical conditions for exclusion included pregnancy at the time the complete blood count was performed, congenital or hereditary anemia, HIV, end-stage kidney disease, cirrhosis, blood cancer, BM transplant and splenectomy. Extreme measures were defined as leukocyte count >200 × 10^9^ l^−1^ or >100 × 10^9^ l^−1^ with 5% immature reticulocytes, hemoglobin concentration >20 g dl^−1^, hematocrit >60%, and platelet count >1,000 × 10^9^ l^−1^. Quality control of these ‘clinical assays’ was done based on methods previously described^41,42^.

### Proteomic profiling

Proteomic profiling was performed in EDTA-plasma samples from ~54,000 UKB participants obtained at baseline as part of the UKB-PPP, using the Olink Explore 1536 and Explore Expansion platforms, which captured 2,923 unique proteins targeted by 2,941 assays. Assay details have been described previously^40,43,44^, including comparisons with seven overlapping clinical assays measured in UKB, yielding strong correlations for matching isoforms (*r* = 0.82)^40^. Briefly, Olink relies on proximity extension assays, which targets proteins by pairs of antibodies conjugated to complimentary oligonucleotides. Upon binding to their target protein, hybridization between probes enables amplification and subsequent relative quantification through next generation sequencing. Protein targeting assays are grouped across four 384-plex panels: inflammation, oncology, cardiometabolic and neurology. Olink’s internal controls involve an incubation (a nonhuman antigen with matching antibodies), extension (IgG conjugated with a matching oligonucleotide pair) and amplification controls (synthetic double-stranded DNA). Additional external controls are included in each plate, namely negative, plate and sample controls. Limit of detection values are calculated for each protein targeting assay per plate based on negative controls run in triplicate. Normalized protein expression (NPX) values are generated by normalization to the extension control, log_2_ transformation and further normalization to the plate controls. Samples are flagged with a warning if NPX values from internal controls are not within ±0.3 NPX from the plate median across an abundance block, or if the mean assay count for a sample is less than 500. Assays are flagged with a warning if the median from the negative control triplicated deviate more than 5 s.d. from predefined values set by Olink. We excluded (1) participants that were removed from the study and (2) samples that were defined as outliers. Outliers included individuals for which standardized first or second principal component values were further than 5 s.d. from the mean or had a median NPX or IQR of NPX greater than 5 s.d. for the mean median or mean IQR. Individual datapoints with sample or assay warnings, or those belonging to 70 plates that failed to satisfy QC criteria were set to missing.

### Incident disease definitions

We developed prediction models for 218 diseases, with more than 80 incident cases within 10 years of follow-up (censoring date was the 31 December 2020 or death date if this occurred first) in the random subset (*N* = 41,931, 193 diseases), or by including incident cases within the ‘consortium-selected’ subset (25 diseases) (Supplementary Table 1). The 218 diseases include common and rare diseases, and diseases associated with high morbidity, high mortality or both. Disease definitions were based on validated phenotypes described by Kuan et al.^14^ by integrating data from primary care available only for a subset of participants (that is, not using any primary care data made available solely for COVID research), hospital episode statistics, cancer and death registries and from UKB health questionnaires, including self-reported illnesses. We excluded prevalent cases (first occurrence before or up to the baseline assessment visit) or incident cases recorded within the first 6 months of follow-up. We note that we did not exclude ‘controls’ (that is, individuals that did not develop the disease under study) with other prevalent conditions. This represents the scenario that is closest to the clinical reality were multimorbidity is increasingly common and the most useful prediction models will be those that can discriminate the outcome of interest in the presence of other underlying diseases or conditions.

We performed a sensitivity analysis for 19 of the 25 diseases, for which incident cases among consortium-selected participants were included. For these 19 diseases, there were at least 60 incident cases within the random subset of UKB-PPP, enabling demonstrating good agreement in predictive performance from the main analyses and by excluding consortium-selected incident cases from the test set (Pearson *r* = 0.97). This showed no strong bias introduced from inclusion of participants who were selected based on specific characteristics or genetic risk of specific diseases.

### Protein and biomarker imputation

After quality control, we imputed missing NPX values, using the missForest R package^45^, for all individuals from the randomized or consortium-selected subsets who met the QC and inclusion criteria, had no missing data for age, sex and BMI, and had no more than 50% of missing values across all proteins (*N* = 48,054; 41,931 from the randomized subset and 6,123 from ‘consortium-selected’ cases; Supplementary Table 2). Imputation was done per panel (that is, separately for Cardiovascular, Cardiovascular II, Inflammation, Inflammation II, Neurology, Neurology II, Oncology and Oncology II panels), including additional information on age and sex. Subsampling (that is, without replacement) was used to grow the number of trees in each forest, which, in turn, was set to 50 (‘ntree’ parameter). As a sensitivity analysis, we tested all optimized models in individuals from the validation set that had no missing values (for the proteins from the final model) to assess the quality of the imputation procedure. We observed good agreement between performance metrics derived in the test set, which included a small proportion of imputed protein values and those derived from individuals with no missing data (Pearson *r* = 0.94).

We further imputed missing values for clinical assays (UKB Category 17518) and nine blood cell traits (leukocyte, lymphocyte, monocyte, neutrophil, eosinophil, basophil, platelet count, hemoglobin concentration and hematocrit percentage) in the individuals who also had clinical assays available (*N* = 47,901).

### Statistical analyses

We adapted a three-step machine learning framework including (1) feature selection, (2) hyperparameter tuning and optimization and (3) validation. Individuals were grouped as follows: 50% for feature selection, 25% for model optimization (training), and 25% for validation, for diseases with more than 800 cases; otherwise, into a 70% feature selection and model optimization set and 30% for validation. Validation sets included nonoverlapping individuals completely blinded to previous model development stages.

We used regularized Cox regression to derive a ‘benchmark’ clinical model, by fivefold crossvalidation in the optimization or training set using the features described above. Validation was performed in the held-out test set, where we computed the C-index over 1,000 bootstrap samples.

For each disease, we performed feature selection among 2,941 protein targets, or among the 37 clinical assays by least absolute shrinkage and selection operator (LASSO) regression over 200 subsamples of the feature selection set. While six proteins were measured across four Olink panels, we included all measurements, albeit for the same protein. This was to enable data-driven selection of the best performing set of measurements given our machine learning framework will shrink coefficients to zero for strongly correlated variables. This also allowed for previously proposed biomarkers to compete with all available proteins in a data-driven framework. In each iteration, we ran fivefold crossvalidation over three repeats using a grid search to tune the hyperparameter lambda, implemented with the caret R package. We used the ROSE R package^46^ to address case imbalance. Selection scores were computed as the absolute sum of weights from the model with the optimal lambda from each of the 200 iterations and were used to identify the top 20 proteins or clinical assays. The top 20 proteins or clinical assays with the highest feature selection scores were taken forward for optimization of a regularized Cox model including the clinical risk factors, by fivefold crossvalidation (optimization set, or feature selection set for diseases with fewer than 800 cases), implemented through the glmnet R package. To further identify sparser predictor sets, the top five and top ten features were identified as those with the highest product of the weights from optimized models (clinical risk factors + top 20 features) and feature selection scores. Optimization of a clinical model plus five or ten features was similarly done by regularized Cox regression by fivefold crossvalidation (optimization set). Performance was tested in the validation set, by computing the C-index over 1,000 bootstrap samples. Finally, models based on the top five proteins alone (without any clinical risk factors) were further trained and tested in the same manner.

We tested improvement in models by adding onto the clinical ‘benchmark’ model: (1) 5–20 proteins, (2) 5–20 clinical assays or (3) genome-wide PGSs^37^ (UKB category 301) (Fig. 1). For these comparisons, we kept the best performing protein signature and clinical assay signature as the one that had the highest C-index in the validation set. Significant improvements between models were considered as those for which the 95% CI of the differences in the bootstrap C-index distributions did not include zero.

We calculated the following screening metrics: DRs and LRs in the validation set at FPR ranging from 5% to 40%. The FPR was calculated as FPR = false positives (FP)/(true negatives (TN) + FP); and detection rates were calculated as DR = true positives (TP)/(false negatives (FN) + TP). LRs were computed as LR = DR/FPR. All analyses were performed in R software v.4.1.1.

We calculated category-free net reclassification improvements from addition of proteins to the clinical models using a 0.15 cut-off in risk difference to provide more conservative estimates, using the R package nricens. We further calculated integrated discrimination improvements from addition of proteins to the clinical models using the R package survIDINRI.

### Age- and sex-stratified performance of prediction models

The performance of the clinical and clinical + protein models was tested by stratifying the validation set by sex (men versus women) and age at onset (<65 years versus ≥65 years at disease onset). We retained only 121 and 134 diseases for which sex-stratified and age-stratified validation sets had at least 20 incident disease cases, respectively. We computed the C-index over 1,000 bootstrap samples of the stratified validation sets. Significant differences between age- or sex-stratified performance were considered as those for which the 95% CI of the differences in the bootstrap C-index distributions did not include zero. Similarly, significant differences between stratified performance of protein-informed models and clinical models were considered as those for which the 95% CI of the differences in the bootstrap C-index distributions did not include zero.

### Performance of prediction models for 5-year incidence

The performance of the clinical and clinical + protein models trained to predict the risk of 10-year incidence, was tested for 5-year incidence (same validation sets). This was tested for diseases for which 10-year incidence prediction (C-index) was significantly improved or improved by more than 4%, and had at least 20 incident cases within 5 years of follow-up in the validation set (54 diseases).

### Predictive performance of the Olink Explore 1536 versus Expansion panels

We further repeated the entire procedure (that is, feature selection, model optimization and testing) on the first subset of Olink Explore 1536 proteins, using the exact same data splits for comparability (that is, the same individuals used in this analysis as those used in training/testing for the main analyses done on 1536 + Expansion proteins).

### Downsampling sensitivity analysis

We performed an additional analysis to rule out the possibility that a statistical artifact could lead to the observed inverse relationship between incident case numbers and the improvement in C-index achieved by proteins. We used hypertension (the disease with the highest number of incident cases) as an example to run this sensitivity analysis, in which we restricted selection of the number of incident cases to 80, 100, 150, 250, 500, 1,000 and 2,000. We repeated the entire framework, including, feature selection, model optimization and validation, in these different configurations including fewer incident cases. We showed there was no inflation in the improvements in C-index achieved by adding proteins onto the clinical model, when restricting the analyses to fewer incident cases (Supplementary Table 13).

### Proportion of variance explained in protein plasma levels

We used the variancePartition R package^47^ to estimate the proportion of variance explained in plasma levels of each of the proteins by a joint model including age, sex, BMI, smoking status and the Elixhauser comorbidity index^48^ as explanatory variables. Briefly, this method fits a linear mixed model and estimates the proportion of variance explained attributed to each of the explanatory variables. We used this framework to identify the main correlates for each of the five proteins. We compared the proportion of variance explained by each of the variables for these five proteins with the average proportion of variance explained across all other proteins.

### Tissue mapping of proteins

To understand the possible tissue origin of plasma proteins, we programmatically downloaded tissue- and cell-type specificity data from the Human Protein Atlas (HPA)^49^ for the Olink proteins in JSON format (on 30 December 2022).

Before joining HPA data with Olink data, we split Olink IDs corresponding to several proteins (protein complexes) into their components based on ENSEMBL gene IDs. Nine proteins (AKR7L, ANP32C, BTNL10, FHIP2A, HCG22, KIR2DL2, KIR2DS4, LILRA3, PNLIPRP2) assayed by Olink were not found on HPA, and NTproBNP was assigned to NPPB, leaving 2,918 unique protein targets.

To determine whether proteins that HPA reports as tissue specific were enriched among selected protein candidates, we performed a two-sided Fisher’s exact test for each tissue-specificity, with the number of selected/nonselected and specific/nonspecific proteins. We defined tissue specific as ‘enhanced,’ or ‘enriched’ according to HPA classification. Some proteins were hence ‘specific’ to several tissues.

### Pathway enrichment

We performed pathway enrichment analysis using the R package gprofiler2 (v.0.2.1)^50^ restricting to KEGG and REACTOME database to maintain specificity. We used all protein coding genes covered by the Olink Explore platform as a background and tested for enrichment of (1) selected protein candidates per disease and (2) proteins selected for at least three diseases. We used the Benjamini–Hochberg (BH) procedure to account for multiple testing.

### MM scRNA-seq analyses

The scRNA-seq data including UMAP representation, cell-type annotation and plasma cell malignancy classification via inferCNV was taken from ref. ^16^. Differential gene expression between BM cell types and healthy versus malignant states was investigated by comparing the mean expression levels of the gene of interest per patient or control using Wilcoxon rank sum test. BH was used to adjust for multiple comparisons.

### External validation

To provide evidence of generalizability of the models developed in UKB, we tested performance of the clinical and protein-informed models in the EPIC-Norfolk study. The EPIC-Norfolk study is a cohort of 25,639 middle-aged individuals from the general population of Norfolk—a county in Eastern England^51^. The study was approved by the Norfolk Research Ethics Committee (reference no. 05/Q0101/191). Participants provided written informed consent.

Participants from the EPIC-Norfolk study^51^ were flagged for mortality at the UK Office of National Statistics and vital status was ascertained for the entire cohort. Death certificates, hospitalization data and cancer registry data was obtained using National Health Service (NHS) numbers through linkage with the NHS digital database. EHRs were coded by trained nosologists according to the International Statistical Classification of Diseases and Related Health Problems, ninth (ICD-9) or tenth Revision (ICD-10). Participants were identified as having experienced an event if the corresponding ICD-10 code was registered on the death certificate (as the underlying cause of death or as a contributing factor), cancer registry or as the cause of hospitalization. Given that the long-term follow-up of EPIC-Norfolk included the ICD-9 and ICD-10 coding system, codes were consolidated.

Serum samples from the baseline assessment (1993–1997) that had been stored in liquid nitrogen were used for proteomic profiling of a randomly selected subcohort (*N* = 749; Supplementary Table 14) and a T2D case-cohort study (*N* = 1,173; Supplementary Table 14), using the Olink Explore 1536 and Olink Explore Expansion panels, targeting 2,923 unique proteins by 2,941 assays. Participants were excluded due to failed proteomic QC, missing information on age, sex, BMI or smoking status.

Out of the 67 diseases for which proteins improved prediction over and above the clinical benchmark in UKB, we were able to test model replication in the EPIC-Norfolk study for T2D (in the T2D case-cohort), prostate cancer, heart failure, COPD, chronic kidney disease and cataracts (in the random subcohort) (Supplementary Tables 14 and 15). Because family history of the disease was not available in EPIC-Norfolk, we trained models in UKB without this variable. We used the weights from the models trained in UKB to evaluate their performance in EPIC-Norfolk. While the models developed in UKB were trained for prediction of 10-year incidence, we tested predictive performance for 10-year and 20-year incidence in EPIC-Norfolk given the low sample size and design of this study. We excluded prevalent cases (for the disease being tested) and incident cases occurring within the first 6 months of follow-up. Performance was tested in EPIC-Norfolk, by computing the C-index over 1,000 bootstrap samples. As in UKB, significant improvements between models were considered as those for which the 95% CI of the differences in the bootstrap C-index distributions did not include zero.

### Reporting summary

Further information on research design is available in the Nature Portfolio Reporting Summary linked to this article.

## Online content

Any methods, additional references, Nature Portfolio reporting summaries, source data, extended data, supplementary information, acknowledgements, peer review information; details of author contributions and competing interests; and statements of data and code availability are available at 10.1038/s41591-024-03142-z.

## Supplementary information

Supplementary Information Supplementary Figs. 1–5.

Reporting Summary

Supplementary Tables 1–22 Supplementary Tables 1–22 and table headers 1–22.

## Data Availability

All proteomic, phenotypic and EHR data used in this study are available from UKB upon application (https://www.ukbiobank.ac.uk). The EPIC-Norfolk data can be requested by bona fide researchers for specified scientific purposes via the study website (https://www.mrc-epid.cam.ac.uk/research/studies/epic-norfolk/). Data will either be shared through an institutional data sharing agreement or arrangements will be made for analyses to be conducted remotely without the need for data transfer. Data from the Human Protein Atlas is publicly available (https://www.proteinatlas.org/). KEGG (https://www.genome.jp/kegg/) and REACTOME (https://reactome.org/) pathway data is also publicly available. scRNA-seq data are available at the European Genome-Phenome Archive under accession number EGAS00001006980. To accelerate the use and translational potential of our findings, we generated an open-access interactive web resource that enables the scientific community to easily visualize post-test probabilities based on derived LRs across all diseases (https://omicscience.org/apps/protpred).

## Code availability

Associated code and scripts for the analysis can be found in the following GitHub repository: https://github.com/comp-med/Sparse-proteomic-prediction-of-common-and-rare-diseases.git.
