# Supplementary material for: Proteomic signatures improve risk prediction for common and rare diseases
Source: Nat Med. 2024 Jul 22;30(9):2489–98. doi: 10.1038/s41591-024-03142-z (PMC11405273; doi:10.1038/s41591-024-03142-z)
Supplement: Supplementary file 1 — Supplementary Figs. 1–5. [file 41591_2024_3142_MOESM1_ESM.pdf]

nature medicine

Supplementary information https://doi.org/10.1038/s41591-024-03142-z

Proteomic signatures improve risk prediction for common and rare diseases

In the format provided by the authors and unedited

Supplementary Figures

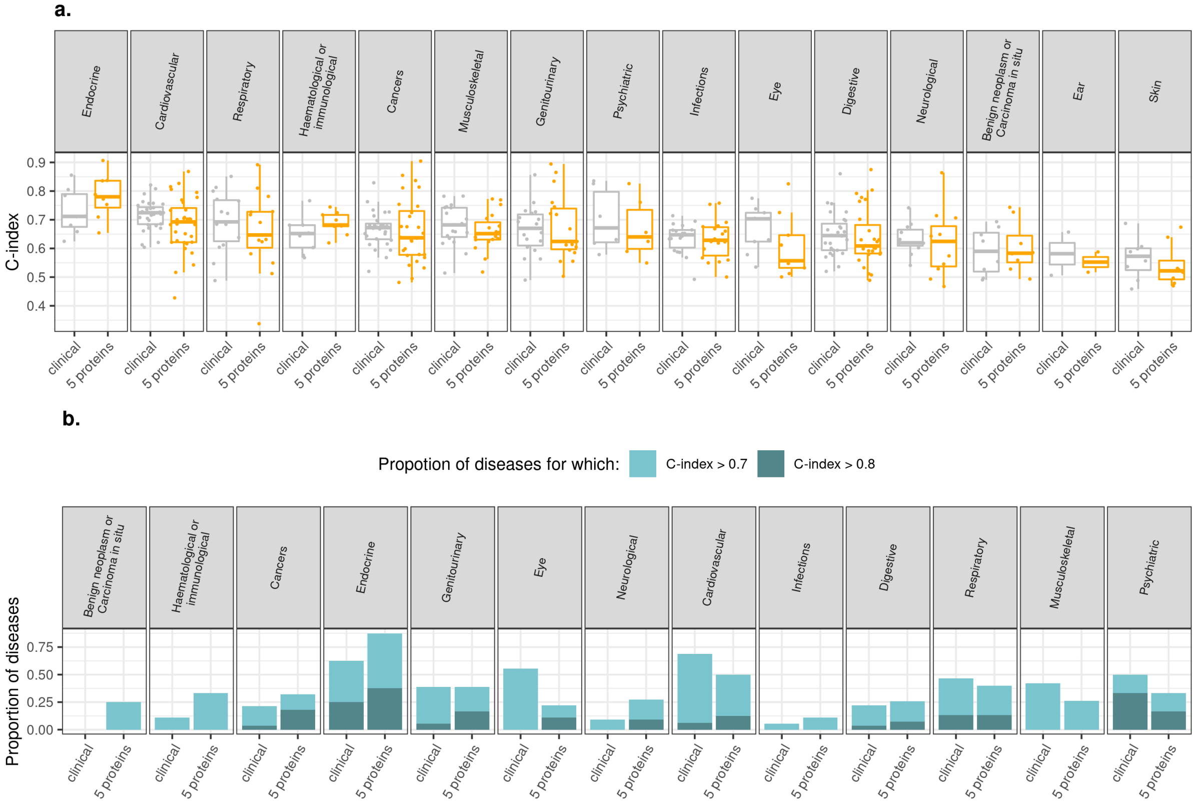

Supplementary Figure 1. Predictive performance of 5 proteins alone compared to the benchmark clinical models in the test set. a, Distribution of the predictive performance (C-index) achieved by 5 proteins (orange) and clinical risk factors (grey) within each disease category. Data are presented as median values; box edges are 1st and 3rd quartiles; and whiskers represent 1.5× interquartile range (N = 218 diseases). **b,** Proportion of diseases within each category for which the clinical risk factors or the top 5 proteins achieved a C-index > 0.7 (full bar height). Bar heights coloured in darker blue represent the proportion of diseases for which the C-index >0.8.

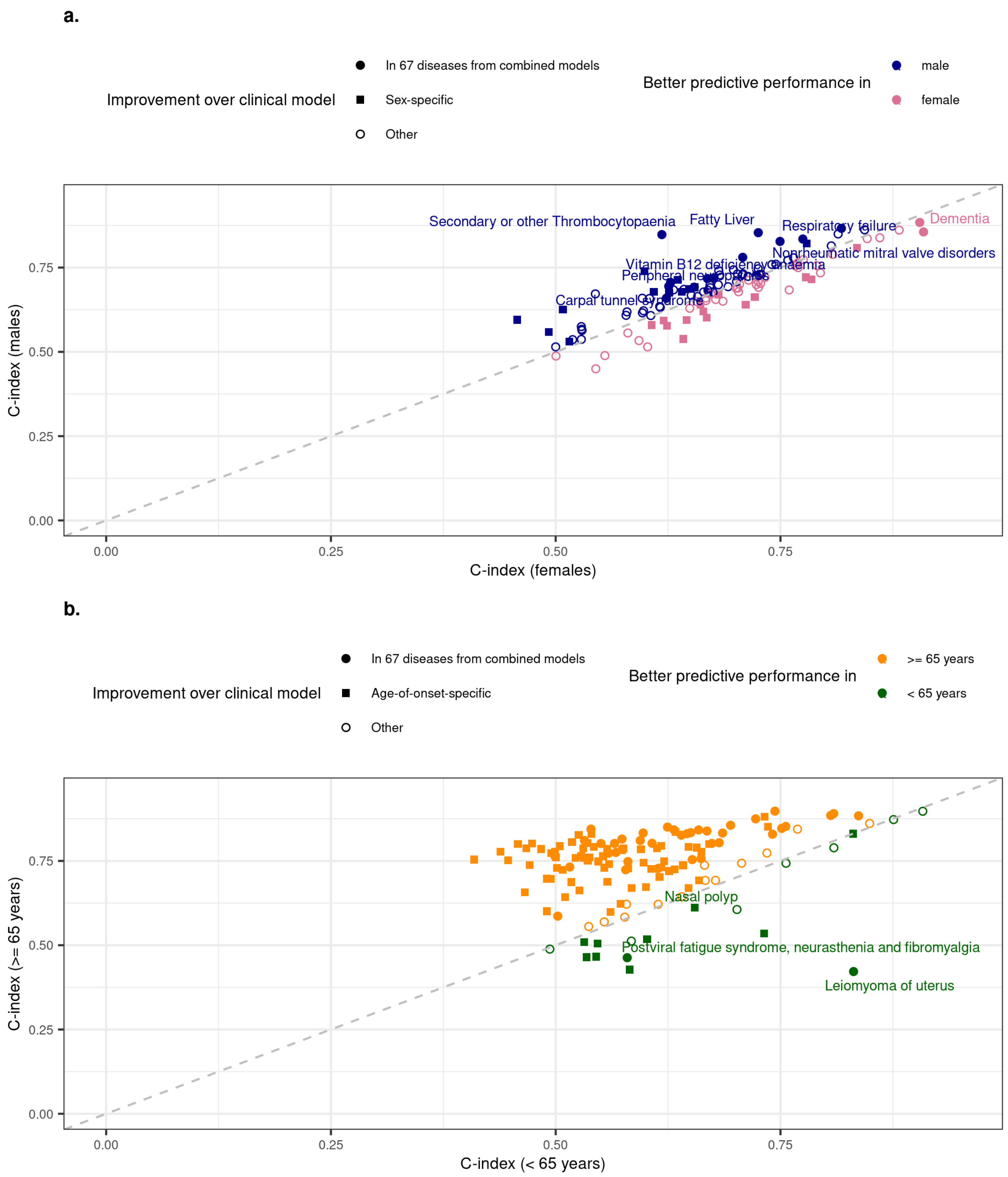

Supplementary figure 2. Comparison of clinical + protein model performance in sex and age of onset strata of the validation set. a, Comparison of C-index in men and women. **b,** Comparison of C-index for discrimination of younger age at disease onset (<65-year) vs older age at onset (>=65 years). Only diseases with evidence of significantly different performance between the two strata are shown. Filled points represent examples for which the protein model significantly improved prediction over the clinical model.

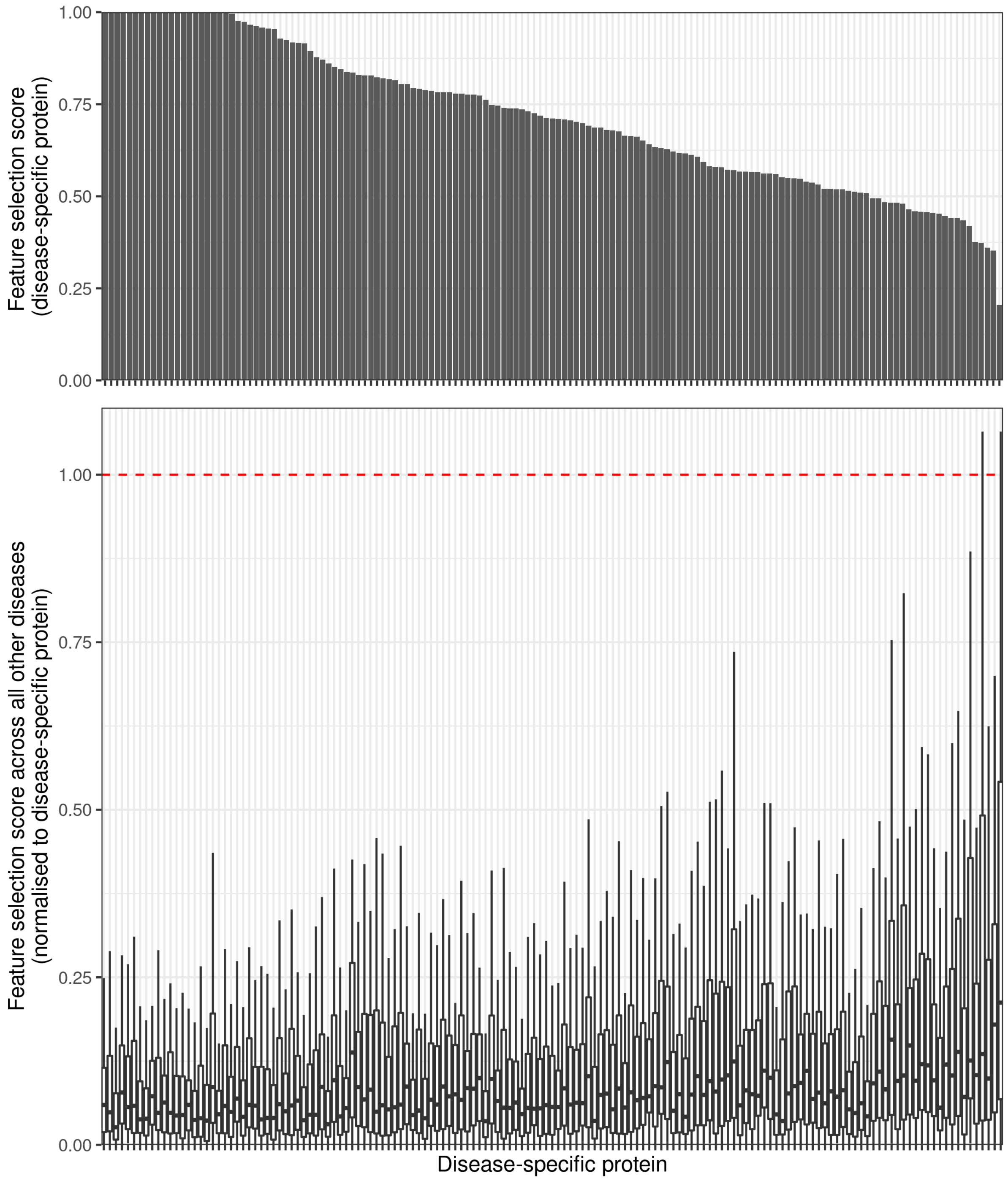

Supplementary figure 3. Feature selection scores for proteins defined as disease-specific. Top – feature selection score for the corresponding disease is shown for each of the disease-specific proteins. Bottom – Distribution of feature selection scores for proteins defined as specific, across all other diseases that were improved by protein signatures. Feature selection scores are normalised to the score of the specific disease. Data are presented as median values; box edges are 1st and 3rd quartiles; and whiskers represent 1.5× interquartile range.

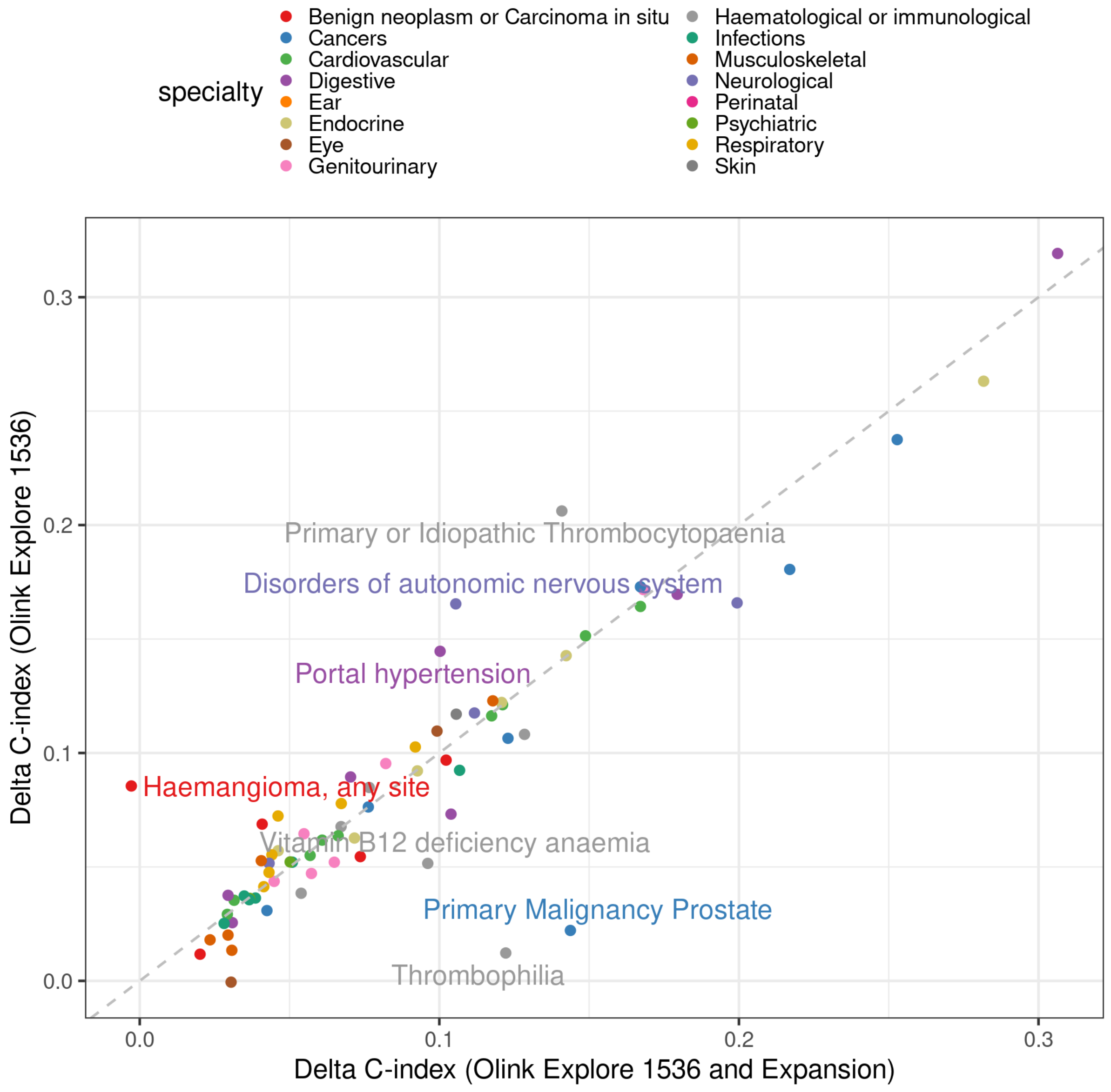

Supplementary figure 4. Comparison of improvement in predictive performance provided from proteins derived from the Explore 1536 + Expansion and Explore 1536 platforms. Comparison is shown for those diseases which were significantly improved by proteins over the clinical model for either analysis.

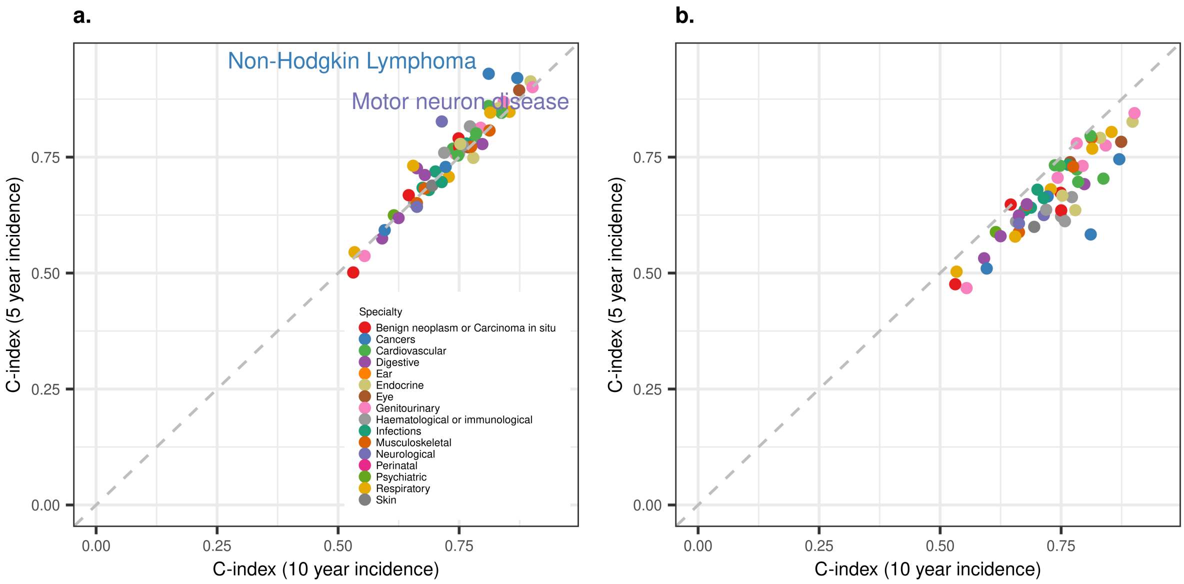

Supplementary figure 5. Predictive performance of 10 year incidence models for 5 year incidence. a, Protein-based models trained for prediction of 10 year incidence were tested for 5 year incidence prediction. This analysis was restricted to diseases which were improved by proteins over the clinical risk factors (or improved the C-index by more than 5%), and had at least 20 incident cases during 5 years of follow-up in the test set (54 diseases). **b,** Clinical information (age, sex, BMI, self-reported ethnicity, smoking status, alcohol consumption and paternal of maternal history of disease) models trained for prediction of 10 year incidence were tested for 5 year incidence prediction for the same 54 diseases.
