# Supplementary material for: Proteomic signatures improve risk prediction for common and rare diseases
Source: Nat Med. 2024 Jul 22;30(9):2489–98. doi: 10.1038/s41591-024-03142-z (PMC11405273; doi:10.1038/s41591-024-03142-z)
Supplement: Supplementary file 2 — Reporting Summary [file 41591_2024_3142_MOESM2_ESM.pdf]

Corresponding author(s): Claudia Langenberg
Last updated by author(s): Mar 8, 2024

Reporting Summary

Nature Portfolio wishes to improve the reproducibility of the work that we publish. This form provides structure for consistency and transparency in reporting. For further information on Nature Portfolio policies, see our Editorial Policies and the Editorial Policy Checklist.

Statistics

For all statistical analyses, confirm that the following items are present in the figure legend, table legend, main text, or Methods section.

n/a	Confirmed	
☐	☒	The exact sample size (*n*) for each experimental group/condition, given as a discrete number and unit of measurement
☐	☒	A statement on whether measurements were taken from distinct samples or whether the same sample was measured repeatedly
☐	☒	The statistical test(s) used AND whether they are one- or two-sided<br>*Only common tests should be described solely by name; describe more complex techniques in the Methods section.*
☐	☒	A description of all covariates tested
☐	☒	A description of any assumptions or corrections, such as tests of normality and adjustment for multiple comparisons
☐	☒	A full description of the statistical parameters including central tendency (e.g. means) or other basic estimates (e.g. regression coefficient) AND variation (e.g. standard deviation) or associated estimates of uncertainty (e.g. confidence intervals)
☐	☒	For null hypothesis testing, the test statistic (e.g. *F*, *t*, *r*) with confidence intervals, effect sizes, degrees of freedom and *P* value noted<br>*Give P values as exact values whenever suitable.*
☒	☐	For Bayesian analysis, information on the choice of priors and Markov chain Monte Carlo settings
☒	☐	For hierarchical and complex designs, identification of the appropriate level for tests and full reporting of outcomes
☐	☒	Estimates of effect sizes (e.g. Cohen's *d*, Pearson's *r*), indicating how they were calculated

Our web collection on statistics for biologists contains articles on many of the points above.

Software and code

Policy information about availability of computer code

Data collection	No software was used for data collection in this study.
Data analysis	R (v4.1.1)<br>glmnet R package 4.1-2<br>caret R package 6.0-88<br>ROSE R package 0.0-4<br>missForest R package 1.4<br>nricens R package 1.6<br>survIDINRI R package 1.1-1<br>variancePartition R package 1.22.0<br>gprofiler R package v0.2.1<br>We have deposited the code used for this study in the folloeing GitHub repository: https://github.com/comp-med/Sparse-proteomic-prediction-of-common-and-rare-diseases.git

For manuscripts utilizing custom algorithms or software that are central to the research but not yet described in published literature, software must be made available to editors and reviewers. We strongly encourage code deposition in a community repository (e.g. GitHub). See the Nature Portfolio guidelines for submitting code & software for further information.

Data

Policy information about availability of data

All manuscripts must include a data availability statement. This statement should provide the following information, where applicable:

- Accession codes, unique identifiers, or web links for publicly available datasets
- A description of any restrictions on data availability
- For clinical datasets or third party data, please ensure that the statement adheres to our policy

All proteomic, phenotypic and EHR data used in this study are available from the UK biobank upon application (https://www.ukbiobank.ac.uk). The EPIC-Norfolk data can be requested by bona fide researchers for specified scientific purposes via the study website (https://www.mrc-epid.cam.ac.uk/research/studies/epic-norfolk/). Data will either be shared through an institutional data sharing agreement or arrangements will be made for analyses to be conducted remotely without the need for data transfer. Data from the Human Protein Atlas is publicly available (https://www.proteinatlas.org/). KEGG (https://www.genome.jp/kegg/) and REACTOME (https://reactome.org/) pathway data is also publicly available. Single-cell RNA sequencing data are available at the European Genome-Phenome Archive (EGA) under accession number EGAS00001006980.

Research involving human participants, their data, or biological material

Policy information about studies with human participants or human data. See also policy information about sex, gender (identity/presentation), and sexual orientation and race, ethnicity and racism.

Reporting on sex and gender	This study included both males and females that met inclusion/ exclusion criteria. Sex of the participants was based on self-report and included as a predictor variable in all models..
Reporting on race, ethnicity, or other socially relevant groupings	This study included participants form all ethnicities that met inclusion/exclusion criteria. Ethnicity of participants was based on self-report and included as a predictor variable in all models.
Population characteristics	UK Biobank comprises up to 502,650 participants aged between 40 to 69 years at baseline recruited across 22 assessment centres in England, Scotland and Wales. The average age at baseline was 56.52 years (standard deviation, SD 8.09). Of the 502,650 volunteers, 273,468 were women (54.41%), who were on average younger than the men (56.35 years, SD 8.00). Additional details are provided in Hewitt et al. (BMJ Open, 2016).<br>A comparison of the UK Biobank with individuals in the general population, conducted by Fry et al. (American Journal of Epidemiology, 2017) found that UKB participants were, "more likely to be older, to be female, and to live in less socioeconomically deprived areas than nonparticipants", suggesting evidence of a selection bias towards healthy volunteers.<br>EPIC-Norfolk participants selected for the subcohort were on average 58.60 years old (standard deviation: 9.34). EPIC-Norfolk cases selected for the case-cohorts were on average 59.44 years old (standard deviation: 9.25).
Recruitment	The recruitment strategy for UK Biobank is described in detail by Bycroft et al (Nature, 2018). Briefly, participants aged 40 to 69 years were recruited across the United Kingdom between the years 2006 and 2010 from the National Health Service (NHS) patient registers. Approximately 9.2 million people living 25 miles (40 km) from one of 22 assessment centers across England, Wales and Scotland were invited to participate, with 5.5% participating in the baseline studies. All participants completed self-report questionnaires detailing their demographic, socioeconomic and health-related characteristics. Participants also underwent several physical assessments (e.g., repeated blood pressure measurements, weight and height). Participants also provided blood, urine and saliva samples, which were then stored in a central storage facility in Stockport, United Kingdom.<br>The EPIC-Norfolk study is a cohort of 25,639 middle-aged individuals from the general population of Norfolk, a county in Eastern England.
Ethics oversight	Ethics approval for the UK Biobank study was obtained from the North West Centre for Research Ethics Committee (11/NW/0382). For this study, access to UK Biobank was approved by the Access Subcommittee of UK Biobank, under Access Management System Application No. 65851 and 20361. The study was approved by the Norfolk Research Ethics Committee (ref. 05/Q0101/191).

Note that full information on the approval of the study protocol must also be provided in the manuscript.

Field-specific reporting

Please select the one below that is the best fit for your research. If you are not sure, read the appropriate sections before making your selection.

☒ Life sciences ☐ Behavioural & social sciences ☐ Ecological, evolutionary & environmental sciences

For a reference copy of the document with all sections, see nature.com/documents/nr-reporting-summary-flat.pdf

Life sciences study design

All studies must disclose on these points even when the disclosure is negative.

Sample size	44,345 participants from the UKB-PPP. We used only sample from the randomly selected subset of UKB-PPP to avoid any biases introduced by "consotrium-selected" samples which where enriched in multiple prevalent diseases. For 25 diseases which had less than 80 incident cases

	within 10-years of follow-up in the randomly selected subset, we included "consotrium-selected" individuals who were incident cases for the disease under study within 10 years of follow-up. This is the largest proteomic study in the world to date.
Data exclusions	Exclusion as part of proteomic QC or due to missing data for basic demographic information (age, sex or BMI) have been described in detail in the methods and supplementary information. For the analysis of each disease, we further excluded participants defined as prevalent cases or with an incident event for the disease within the first 6 months of follow-up
Replication	External replication was performed successfully in the EPIC-Norfolk study for all 6 diseases with sufficient sample size; in 1922 participants (a random sub-cohort of 749 participants; a T2D case-cohort of 1173 participants).
Randomization	This study is based on a randomly selected subset of individuals from the UKB-PPP (described in detail in Sun. B et al. Nature 2023). For 25 out of the 218 diseases under study, we additional included incident cases only from the "consortium-selected" set of participants from UKB-PPP., which were selected based on specific diseases of interest to the Pharma Partners and are therefore enriched in a number of prevalent conditions. We included common clinical covariates in our models such as age, sex, body mass index, smoking status, alcohol consumption, ethnicity and paternal or maternal history of the disease (where available).
Blinding	Blinding does not apply since this is an observational study. Information on protein levels and disease status was needed to perform analyses.

Reporting for specific materials, systems and methods

We require information from authors about some types of materials, experimental systems and methods used in many studies. Here, indicate whether each material, system or method listed is relevant to your study. If you are not sure if a list item applies to your research, read the appropriate section before selecting a response.

Materials & experimental systems

n/a	Involved in the study
☐	☒ Antibodies
☒	☐ Eukaryotic cell lines
☒	☐ Palaeontology and archaeology
☒	☐ Animals and other organisms
☒	☐ Clinical data
☒	☐ Dual use research of concern
☒	☐ Plants

Methods

n/a	Involved in the study
☒	☐ ChIP-seq
☒	☐ Flow cytometry
☒	☐ MRI-based neuroimaging

Antibodies

Antibodies used	A full list of proteins measured using the antibody-based Olink Explore 3072 is provided on the Olink website: https://olink.com/products-services/explore/ All assays in Olink's panels use antigen affinity-purified polyclonal or monoclonal antibodies (or combinations of both), with the majority being commercially available.
Validation	Validation data for the Explore 3072 assay are available on the Olink website: https://olink.com/products-services/explore/

Plants

Seed stocks	*Report on the source of all seed stocks or other plant material used. If applicable, state the seed stock centre and catalogue number. If plant specimens were collected from the field, describe the collection location, date and sampling procedures.*
Novel plant genotypes	*Describe the methods by which all novel plant genotypes were produced. This includes those generated by transgenic approaches, gene editing, chemical/radiation-based mutagenesis and hybridization. For transgenic lines, describe the transformation method, the number of independent lines analyzed and the generation upon which experiments were performed. For gene-edited lines, describe the editor used, the endogenous sequence targeted for editing, the targeting guide RNA sequence (if applicable) and how the editor was applied.*
Authentication	*Describe any authentication procedures for each seed stock used or novel genotype generated. Describe any experiments used to assess the effect of a mutation and, where applicable, how potential secondary effects (e.g. second site T-DNA insertions, mosiacism, off-target gene editing) were examined.*
